# Acute and persistent responses after H5N1 vaccination in humans

**DOI:** 10.1016/j.celrep.2024.114706

**Published:** 2024-09-04

**Authors:** Richard Apps, Angélique Biancotto, Julián Candia, Yuri Kotliarov, Shira Perl, Foo Cheung, Rohit Farmer, Matthew P. Mulè, Nicholas Rachmaninoff, Jinguo Chen, Andrew J. Martins, Rongye Shi, Huizhi Zhou, Neha Bansal, Paula Schum, Matthew J. Olnes, Pedro Milanez-Almeida, Kyu Lee Han, Brian Sellers, Mario Cortese, Thomas Hagan, Nadine Rouphael, Bali Pulendran, Lisa King, Jody Manischewitz, Surender Khurana, Hana Golding, Robbert G. van der Most, Howard B. Dickler, Ronald N. Germain, Pamela L. Schwartzberg, John S. Tsang

**Affiliations:** 1 NIH Center for Human Immunology, NIH, Bethesda, MD 20892, USA; 2 Biometric Research Program, Division of Cancer Treatment and Diagnosis, NCI, NIH, Rockville, MD, USA; 3 Multiscale Systems Biology Section, Laboratory of Immune System Biology, National Institute of Allergy and Infectious Diseases, NIH, Bethesda, MD 20892, USA; 4 NIH Oxford-Cambridge Scholars Program, Cambridge Institute for Medical Research and Department of Medicine, University of Cambridge, UCB2 0QQ Cambridge, UK; 5 Institute for Immunity, Transplantation and Infection, School of Medicine, Stanford University, Stanford, CA 94305, USA; 6 Hope Clinic of the Emory Vaccine Center, Decatur, GA 30030, USA; 7 Division of Viral Products, Center for Biologics Evaluation and Research (CBER), FDA, Silver Spring, MD 20993 USA; 8 GSK, Rixensart, Belgium; 9 Lymphocyte Biology Section, Laboratory of Immune System Biology, National Institute of Allergy and Infectious Diseases, NIH, Bethesda, MD 20892, USA; 10 Cell Signaling and Immunity Section, Laboratory of Immune System Biology, National Institute of Allergy and Infectious Diseases, NIH, Bethesda, MD 20892, USA; 11 Center for Systems and Engineering Immunology, Departments of Immunobiology and Biomedical Engineering, Yale University, New Haven, CT 06520, USA; 12 These authors contributed equally; 13 Lead contact

## Abstract

To gain insight into how an adjuvant impacts vaccination responses, we use systems immunology to study human H5N1 influenza vaccination with or without the adjuvant AS03, longitudinally assessing 14 time points including multiple time points within the first day after prime and boost. We develop an unsupervised computational framework to discover high-dimensional response patterns, which uncover adjuvant- and immunogenicity-associated early response dynamics, including some that differ post prime versus boost. With or without adjuvant, some vaccine-induced transcriptional patterns persist to at least 100 days after initial vaccination. Single-cell profiling of surface proteins, transcriptomes, and chromatin accessibility implicates transcription factors in the erythroblast-transformation-specific (ETS) family as shaping these long-lasting signatures, primarily in classical monocytes but also in CD8^+^ naive-like T cells. These cell-type-specific signatures are elevated at baseline in high-antibody responders in an independent vaccination cohort, suggesting that antigen-agnostic baseline immune states can be modulated by vaccine antigens alone to enhance future responses.

## In brief

Apps et al. use systems immunology to assess H5N1 vaccination with and without the AS03 adjuvant, revealing dynamic response patterns associated with the adjuvant and vaccine immunogenicity. Independent of AS03, some early responses persisted to 100 days after vaccination. These persistent signatures evaluated pre-vaccination predict vaccine immunogenicity in another cohort.

## Graphical Abstract

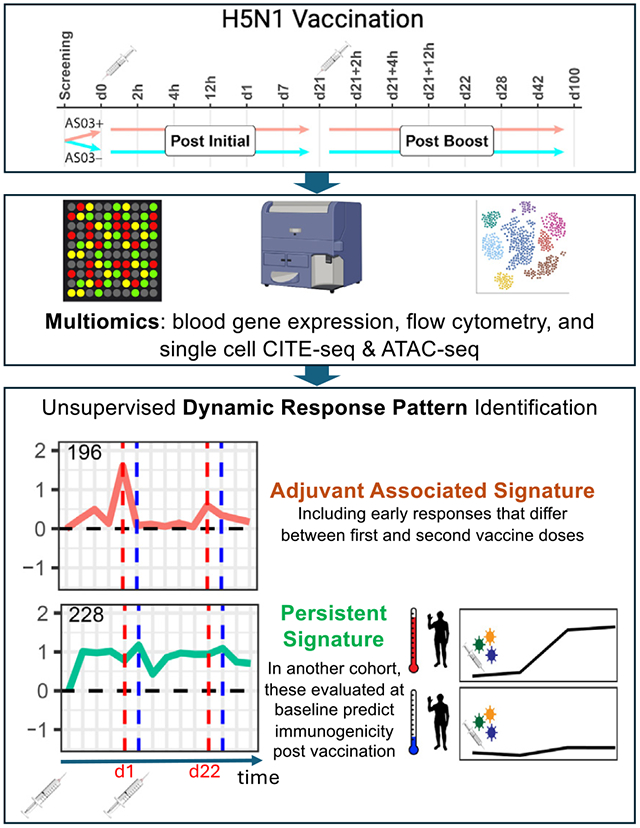

## INTRODUCTION

Vaccines rely on two different properties to produce appropriate immune responses in the host.^1–3^ The first is antigenicity—having the right molecular components to evoke useful adaptive immune responses by T and B lymphocytes that target one or more components of the pathogen, leading to protection against infection or amelioration of disease severity. The second is immunogenicity—the capacity to stimulate cells of the immune system to produce a defense response rather than ignore or even develop tolerance to vaccine antigens. This second activity is typically enhanced in vaccines by components termed adjuvants. The most widely used adjuvant is alum, but more recently, more potent materials have been incorporated into vaccines used in humans, many composed of a lipid and a second substance that together markedly boost antibody and/or T cell responses.

Beyond improved antigen delivery to antigen-presenting cells and stimulation of pattern recognition receptors to induce more robust innate responses, the precise mechanism of action of many adjuvants remains unclear, including how they affect components of the human immune system over different timescales. The AS series of adjuvants, including the AS03 being studied in this work, are potent additions to vaccine antigens that are increasing in use in humans,^4^ making further investigation of their mode of action particularly valuable. Mechanistic studies, predominantly carried out in mice, have shown that AS03 increases migration and antigen uptake by antigen-presenting cells.^5,6^ After administration of the AS03-H5N1 pandemic influenza vaccine, human cellular response studies showed activation of FcγR, IP-10, and antigen-presentation-related genes most prominently at day 1, with increased antigen-specific CD4^+^ T cells expressing CD40L and interleukin-2 (IL-2) present at 21 days after boost vaccination^7–10^ compared to the unadjuvanted formulation. Transcriptomic analysis of responses to other AS adjuvants, such as AS01, has shown inflammatory responses peaking at similar early time points using malaria, tuberculosis, and hepatitis B antigen formulations.^11–13^ Hepatitis B antigen given in combination with different AS adjuvants (AS01, AS03, or AS04) led to transcriptional responses that revealed significant interindividual variation and showed that a defining feature of adjuvant effectiveness *in vivo* is the proportion of recipients in which a core inflammatory signature can be induced.^14^

Given these existing findings, further insight into how AS adjuvants alter immune cell function, and how interindividual variation in response dynamics impacts vaccine responses more broadly, would be of clear value. To address these questions, we have employed a systems immunology approach to assess the dynamic response to a low-antigen-dose, adjuvanted vaccine developed for pandemic preparedness for H5N1 influenza virus, together with a matching control arm in which only the antigen-only vaccine was given without the adjuvant. H5N1 is an avian virus with demonstrated human transmission in 1997 associated with high mortality.^15^ In contrast to seasonal influenza, in which major variation results from viral antigenic drift, new strains such as H5N1 may arise due to viral reassortment that allows strains endemic in animal hosts to become capable of efficient human transmission. Due to its increasingly large avian reservoir and significant ongoing mutations, H5N1 may become capable of efficient human-to-human transmission and thus has been a major focus of pandemic vaccine development.^16^ A current H5N1 vaccine formulation contains a dose-sparing antigen formulation (3.75 μg) of inactivated split-virion influenza strain A/Indonesia/05/2005 adjuvanted with AS03, which is an AS adjuvant containing DL-α-tocopherol and squalene in an oil-in-water emulsion.^17,18^ The AS03 adjuvant is well documented to improve immunogenicity and reduce the amount of antigen needed to elicit a protective response, enabling antigen sparing for greater production capacity during a pandemic.^19–21^ In the context of H5N1 vaccination, the AS03 adjuvant has been described to pro-foundly enhance affinity maturation, with the induced antibody epitope repertoire demonstrating expanded breadth of neutralization against heterologous clades of H5N1.^17,18,21–23^

Here, we report findings from a high-dimensional systems immunology analysis of healthy adults ranging from 22 to 44 years of age who received the H5N1 vaccine in the presence or absence of the AS03 adjuvant. Subjects were sampled intensively over 14 time points out to day 100, including multiple time points within the first 24 h following the primary vaccination and after the boost, to assess early response kinetics. Taking advantage of the dense time sampling and high-dimensional dataset, we developed a computational framework to uncover, blinded to adjuvant status, dynamic “response patterns” (RPs) that enabled quantitative dissection of interindividual response variations. Scoring of subject-level responses enabled identification of adjuvant signatures and predictive parameters of vaccination responses with or without adjuvant.

Unexpectedly, we also uncovered evidence that two-dose (but not single-dose) antigen-sparing vaccinations with or without adjuvant can result in cell-type-specific gene expression patterns that remain altered 100 days after the first vaccine dose. We characterized these long-term effects at single-cell resolution using CITE-seq, uncovering differences most prominently in classical CD14^+^ monocytes and CD8^+^ naive-like T cells. We also profiled chromatin accessibility changes via single-cell ATAC-seq (scATAC-seq), which implicated transcription factors (TFs) that mediate these persistent responses. Functionally, we show that these signatures were elevated at the baseline (pre-vaccination) of individuals who responded with higher antibody titers in an independent study of seasonal and H1N1 vaccination. Thus, natural variation in baseline immune status in the human population may recapitulate similar immune cell states inducible by twice-given antigen-sparing, non-adjuvanted, low-inflammation vaccination, suggesting that baseline immune states can be modulated by an external agent to potentially enhance future responses in an antigen-agnostic manner. Together, our findings provide novel insights into the functional impact of vaccination with and without the AS03 adjuvant on the human immune system and suggest that even a dose-sparing vaccine without adjuvant can bring the immune system to a new baseline set point associated with enhanced future responses.

## RESULTS

### Study design with an analysis plan initially blinded to adjuvant status

**Figure 1.**
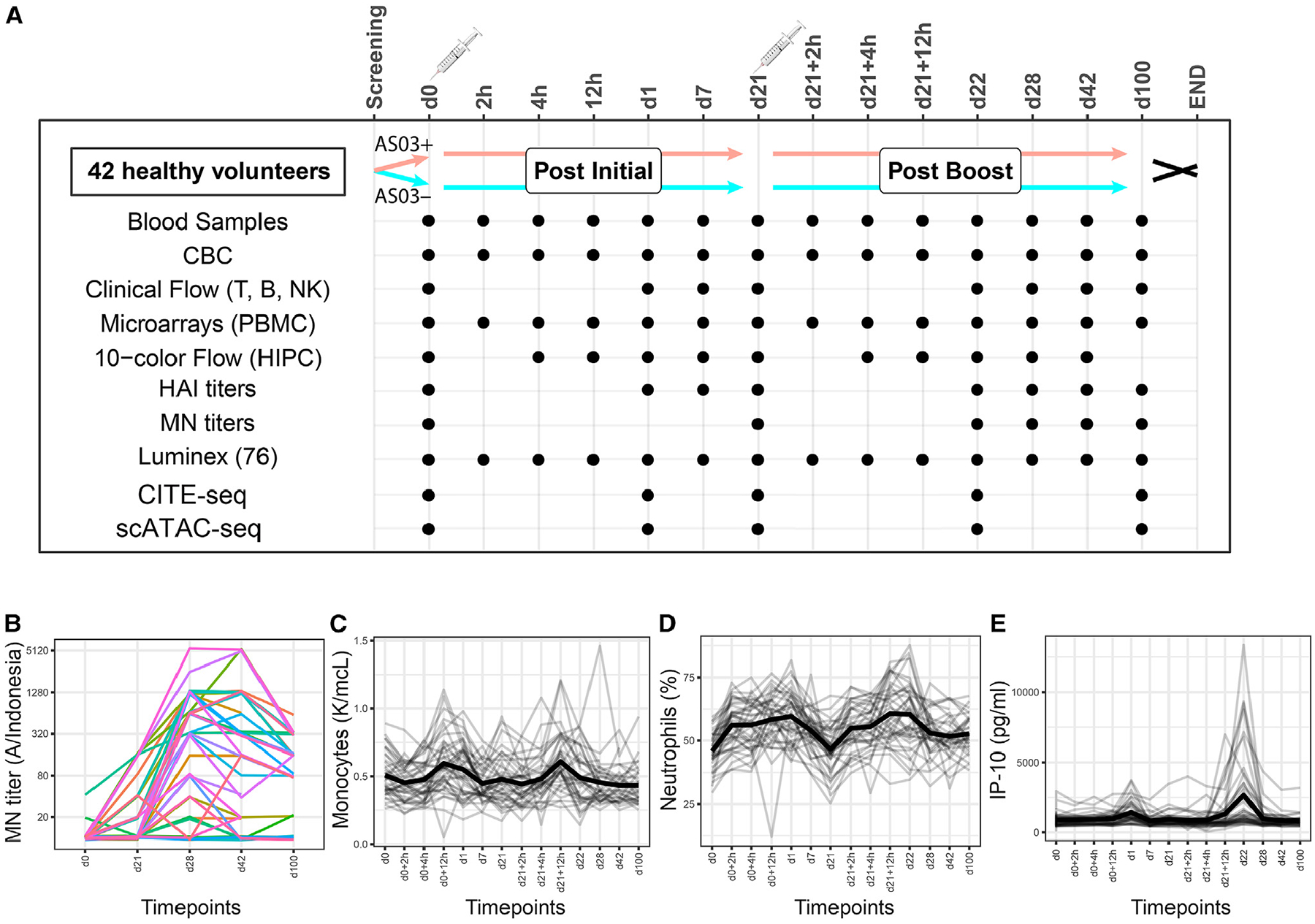
Study design and monitoring of known vaccine response markers (A) A schematic of the study design indicating the pre- and post-vaccination blood collections and the specific assays performed at each time point. Each subject was vaccinated on day 0 and day 21 (boost). A dot represents a time point on which a particular assay was performed. For more detailed information see Table S1. (B–E) Known markers of vaccine response that were monitored throughout the study. Microneutralization (MN) H5N1 titer responses to the vaccine are shown for all subjects in log_2_ scale. Total monocyte concentrations or neutrophil percentages from complete blood count (CBC) tests, or IP-10 concentration determined by Luminex, are shown for all subjects, with mean values marked in bold.

To assess dynamic RPs after vaccination, we enrolled 42 healthy individuals, each of whom received two doses of H5N1 influenza vaccine 21 days apart. Twenty-two subjects received the AS03 adjuvanted vaccine and 20 received the vaccine alone without AS03. Data were kept blinded to the adjuvant status of the subjects during data generation and the first stage of analysis to enable assessment of the response dynamics and adjuvant effects in an unbiased manner. Blood was sampled at 14 time points, including baseline before vaccination and at multiple time points during the first 24 h after each vaccination dose, as well as out to day 100 to assess the initiation of responses and their long-term resolution. Multimodal measurements characterizing cell populations, gene expression, and serum proteins were performed at multiple time points, as well as analyses of serum antibody titers (Figure 1A; Table S1).

Antibody response, measured by microneutralization (MN) titers to H5N1 strain A/Indonesia/5/2005, peaked for most subjects around day 28 (7 days after the second dose), although six individuals likely belonging to the non-adjuvanted group showed no serological increase (Figure 1B). Canonical immune and vaccine response parameters demonstrated dynamical patterns of responses with substantial variability across subjects. Whereas monocytes peaked around 12 h after both doses, neutrophils started to rise as early as 2 h after both doses and returned to baseline after the first but not the second dose; neutrophils remained elevated after the second dose, even at day 100 (Figures 1C and 1D). Circulating IP-10, a protein induced by type I interferons (IFNs) and known to peak on day 1 after vaccination, showed greater responses after the second dose, with highly variable peak magnitudes among subjects, as well as some individuals with no response, who were likely in the non-adjuvanted arm^24,25^ (Figure 1E). These observations suggested that different combinations of immune parameters, comprising transcripts, cytokines, and cell population frequencies, may follow distinct response dynamics and that these dynamic RPs may be specific to particular subsets of individuals due to factors such as whether adjuvant was received, sex, and baseline immune status.

Thus, we devised a data analysis approach that first aimed to uncover, in an unsupervised fashion blinded to the adjuvant status of the vaccinees, dynamic RPs involving combinations of parameters and subjects. Once such “canonical” RPs were identified, we could then transform the high-dimensional dynamic response, involving tens of thousands of parameters over 14 time points, into a compressed set of quantitative response scores for each subject based on how their individual responses concurred with each of the RPs. This would in turn allow linking of subject-level, individual dynamic responses to adjuvant status and antibody responses.

### Unsupervised dynamic response pattern discovery

**Figure 2.**
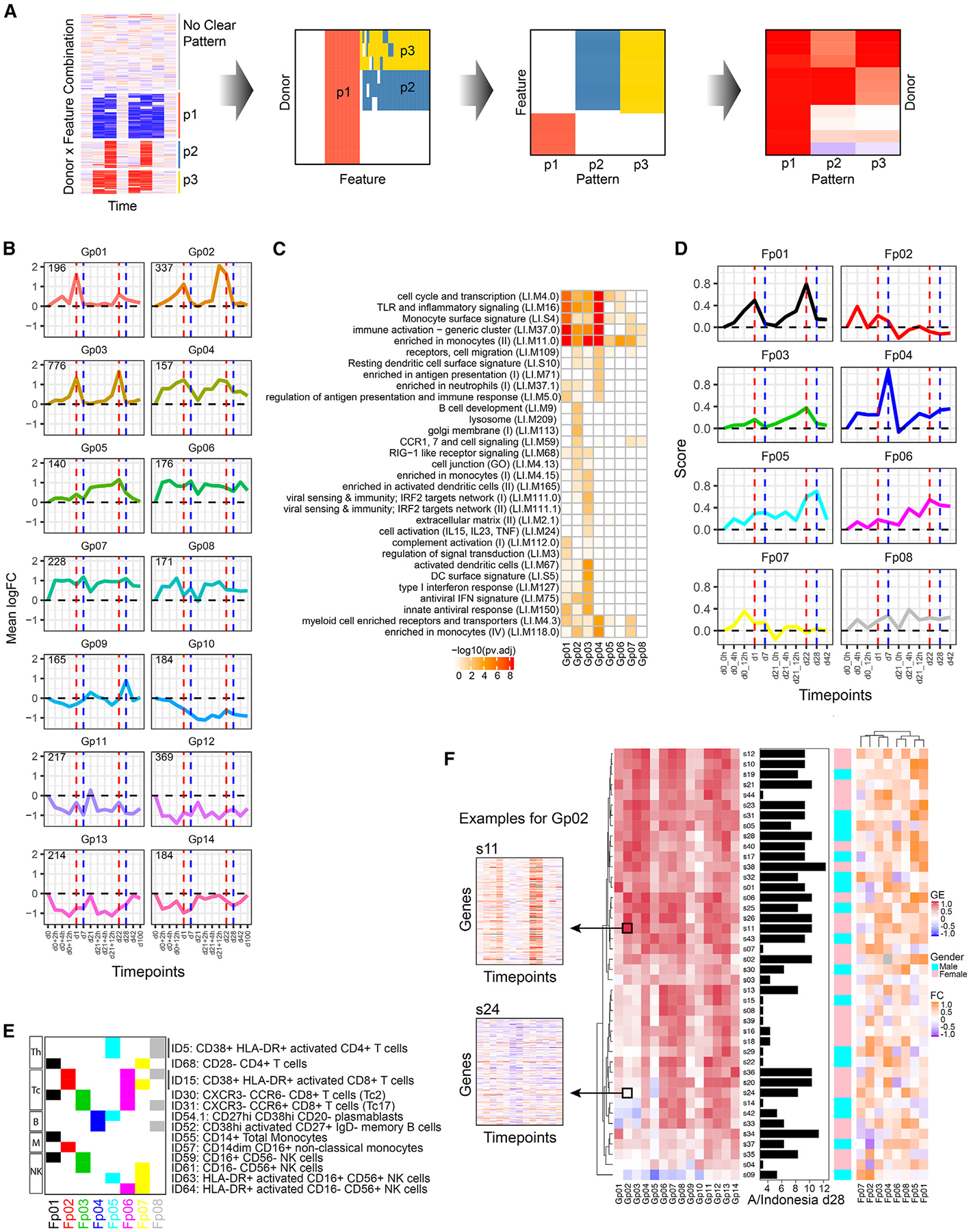
Discovery of high-dimensional dynamic response patterns using gene expression and flow cytometry data (A) A methodological schematic depicting response pattern (RP) discovery. In the leftmost image, patterns are extracted from a matrix where columns are time points and rows are subject and parameter/feature combinations (e.g., transcript level in subject s01), where features are expression of a gene or frequency of a cell population. The middle images illustrate that patterns (p) can be inferred by searching for correlated features present in distinct or overlapping groups of subjects, and once RPs are uncovered, the rightmost image illustrates subject-level (rows) scoring for each pattern (columns). (B) Profiles of the 14 RPs discovered using gene expression data (Gp01 to Gp14). Dashed vertical lines indicate day 1 (red) and day 7 (blue) after initial and boost vaccinations. Numbers on the top left corner of the subplots represent the number of subject-gene combinations comprising each RP profile before the gene lists were expanded using relaxed selection criteria. (C) RPs Gp01 to Gp08 were significantly enriched for the blood transcription modules shown. (D) Profiles of the eight RPs discovered using cell population frequency data (Fp01 to Fp08), with marked vertical lines as described above for (B). (E) The component cell populations for RPs Fp01–08. (F) All 42 subjects clustered by scores for the gene expression RPs Gp01–14. Also shown are MN titers to H5N1 at day 28 (in log_2_ space), subject sex, and cell frequency RP scores (Fp01–08). Expression of the component genes is shown as an example for Gp02 in subjects s11 and s24, individuals with high and low scores for this RP.

We extracted RPs involving blood transcriptomic or cell population frequency parameters as defined by flow cytometry, by searching for multiple parameters with correlated dynamical response trajectories shared among a subset of individuals in the cohort (Figure 2A). Briefly, genes or cell populations were selected with coherent relative changes in a subset of the cohort at any time point after vaccination, the temporal responses of individual subject-gene combinations were then clustered to identify distinct longitudinal patterns, and these RPs were further refined by inclusion of correlated genes or cell populations (see STAR Methods and Figures S1 and S2). This identified 14 transcriptomic (Gp01–14) and 8 cell population (Fp01–08) RPs (Figures 2B–2E).

Among the transcriptomic RPs, several exhibited two peaks that emerged within 24 h after both the primary and the boost vaccinations (Gp01–03), others (Gp04 and 06–08) captured responses that were rapidly developing (within 2 h of vaccination) but were prolonged, with persistent elevation even to day 100, similar to that observed for neutrophil frequency. Further RPs demonstrated decreases in expression, of which some reflected profiles similar to the above RPs but in the opposite (repressed) direction (Gp10–14). Enrichment analysis of blood transcriptomic modules (BTMs) found that some of the RPs reflected cell-type-specific transcriptional signatures, cellular activation, inflammatory states, and other immune functions^26^ (Figure 2C). For example, Toll-like receptor (TLR)/inflammatory signaling and monocyte genes were enriched quite broadly, including Gp01–04; the two-peak patterns (Gp01–03) were enriched for IFN and antiviral response signatures, consistent with early innate response signatures following vaccination.^27–29^ We did not detect significant BTM enrichment for Gp09–14, suggesting genes in these RPs might be less correlated with each other in the human blood transcriptomic datasets used to derive BTMs.^26^

The cell population RPs included patterns similar to those of the transcriptional effects (Fp01, 03, and 05), and others were more unique to the cell population frequency analysis (Fp02, 04, and 06–08) (Figure 2D). The cell populations that comprise each RP showed that monocytes contributed to the two-peak Fp01 and plasmablasts contributed to Fp04 and Fp05, consistent with a plasmablast response 7 days after the second dose as observed for seasonal influenza vaccine (Figures 2D and 2E). Other RPs pointed to potentially novel response elements, including persistent elevation of activated CD8^+^ T cells and natural killer (NK) cells in Fp06 and the presence of CD28^−^CD4^+^ T cells in both Fp01 and Fp07, where some vaccinees had such T cells emerge in the blood after both doses (Fp01), while for others this occurred only after the first dose (Fp07) (Figure S2C).

Together, the transcriptomic and cell frequency RPs uncovered in blinded, unsupervised fashion captured both known and potentially novel vaccine response signatures and provided a basis for investigation of the molecular and cellular determinants of responses to vaccination. We next “scored” the response of each vaccinee against each of the RPs to evaluate the extent to which individual vaccine recipients responded according to the RPs over time (Figure 2F; see STAR Methods). Unsupervised clustering of the subjects based on the gene expression RP scores revealed two major response groups that segregated based on day 28 antibody titers, suggesting that these were associated with adjuvant status given that the AS03 adjuvanted vaccine is known to achieve higher immunogenicity.^17,30^ The main gene expression RPs separating the two groups were the two-peak patterns (Gp01–03), with similar trends observed for cell frequency RPs (Fp01), where plasma-blast-containing patterns were also elevated for individuals with higher antibody titers (Fp05).

### Adjuvant signatures

**Figure 3.**
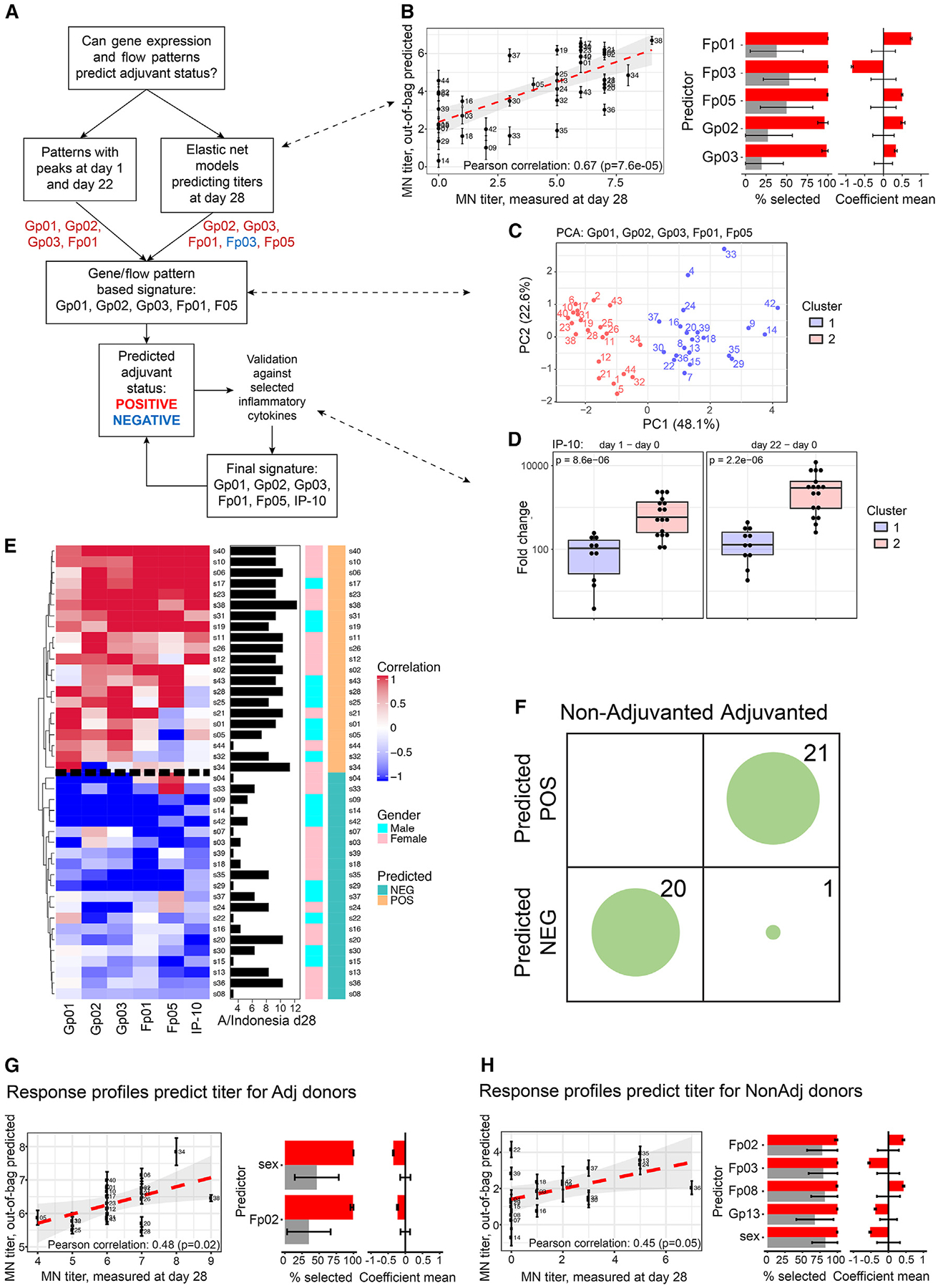
Prediction of adjuvant signature using response patterns identified in blinded analyses and analyses of these patterns as predictors of antibody responses (A) A flowchart of the procedure followed to generate a signature for adjuvant status prediction. (B) Cross-validation-based regularized linear predictive modeling of MN titer responses to H5N1 strain A/Indonesia/5/2005 using elastic net. Input features include response patterns (RPs) discovered from both gene expression and flow cytometry data, sex, and age. Shown are the correlation between out-of-bag predicted titer and actual observed values over multiple iterations of cross validation (see STAR Methods). The top selected features are shown on the right, with the percentage of iterations in which a feature was selected during cross validation and its average coefficient in the linear model. Red bars indicate mean and error bars depict standard deviation across iterations of randomly sampled training and test sets. Gray bars correspond to the result from null model runs in which the same cross-validation procedure was applied to the data with random permutation of sample labels. (C) Principal-component analysis (PCA) of all subjects using the candidate RPs (Gp01, Gp02, Gp03, Fp01, and Fp05) predicted to be associated with the adjuvant based on the approach in (A). Points on the plot are colored according to the two clusters determined by k-means clustering (k = 2). (D) Boxplot of circulating IP-10 responses in the two subject clusters after the primary (day 1–day 0) or boost (day 22–day 0) vaccinations. (E) Heatmap showing RP score profiles. All 42 subjects were clustered by scores for the final adjuvant signature involving Gp01, Gp02, Gp03, Fp01, Fp05, and IP-10. A horizontal dashed black line marks the separation between those predicted to have received the adjuvant (POS) versus not (NEG). MN titer responses to H5N1 at day 28 (in log_2_ space) and sex are also shown. (F) Predicted versus actual adjuvant status shown for all 42 subjects using a confusion matrix. (G and H) Similar to (B), but here, prediction with cross-validation analysis is performed separately for the now unblinded adjuvanted (G) and non-adjuvanted (H) subjects.

We began investigation of the dynamic RPs by identifying those that comprise a signature of the AS03 adjuvant, as this potently increases titer responses. Using a systematic strategy, first the two-peak transcriptomic and cell frequency RPs (Gp01–03 and Fp01) were included, followed by use of an elastic-net and cross-validation-based framework to uncover RPs predictive of the antibody response in the entire set of 42 subjects, as we reasoned that adjuvant status was likely the biggest contributor to antibody response variation. Using as inputs the clearest 12 transcriptomic and 8 cell RPs that we had established, we identified predictive models that significantly forecast day 28 antibody titers to H5N1 strain A/Indonesia/5/2005 (Figure 3B; Figure S3A). The five predictive features included the expected two-peak RPs (Gp02, Gp03, and Fp01) and the plasmablast-containing Fp05 that peaked 7 days following the second dose and also comprised activated NK and CD4^+^ T cells. Fp03 was a negative correlate and thus was not included in our adjuvant signature. Still blinded to adjuvant status, we combined the two-peak RPs and results from predictive modeling to propose an adjuvant signature consisting of Gp01, Gp02, Gp03, Fp01, and Fp05.

Using this proposed adjuvant signature, k-means clustering of the subjects revealed two distinct groups (Figure 3C). Supporting that these reflect adjuvant status, IP-10, a chemokine known to peak on day 1 after vaccination, differed significantly between these two groups in the response to both doses (day 1 – day 0, *p* = 8.6 × 10^−6^; day 22 −day 0, *p* = 2.2 × 10^−6^) (Figure 3D), and similar results were observed for additional inflammatory markers, including CRP, MIP-1b, and SAA (Figure S3B). To help increase the robustness of our proposed signature, we added IP-10 to obtain a final adjuvant signature. Application of this signature to all 42 subjects predicted adjuvanted (POS; “two-peak positive”) and non-adjuvanted (NEG; “two-peak negative”) vaccine recipients, with subjects in the POS group typically showing higher titers (Figure 3E; see STAR Methods). Unblinding the adjuvant status revealed that all 20 of the non-adjuvanted subjects were predicted correctly, and of the 22 adjuvanted subjects, 21 were predicted correctly (Figure 3F). Interestingly, the single adjuvanted subject incorrectly predicted still showed robust two-peak RPs, albeit they were shifted in timing (Figure S4A). The accuracy of adjuvant status prediction was further examined using an independent cohort from Emory University based on our adjuvant-associated gene expression RPs and IP-10 levels (cell frequency data were not available). Adjuvant status was correctly predicted in 32/34 adjuvanted and 14/16 non-adjuvanted subjects (Figures S4B and S4C). Thus, although a small fraction of individuals display RPs that are not typical of their adjuvant status, overall, the signature uncovered by our blinded, unsupervised analysis is a robust reflection of adjuvant status.

Most of the features identified in the adjuvant signature reflect acute innate/inflammatory responses, peaking within 1 day of both vaccination doses. Importantly, enabled by our dense sampling, this signature also revealed differences in the timing, quality, and intensity of responses between the first and the second dose. For example, Gp01 (enriched for monocyte activation) was more elevated after the initial than the second dose, whereas dendritic cell gene expression signatures were prominent at both time points (Gp03). Other signature components appeared to increase more after the second than the first dose (Figures 2B and 2D) and captured broad inflammatory responses, e.g., IFN signatures (IP-10 and Gp02, which also peaked earlier at 12 h after the second vaccine dose) and monocyte frequency (Fp01). Together, the results of our initially blinded adjuvant signature discovery approach confirmed known adjuvant-associated parameters and pointed to response dynamic and early-response differences between the first and the second vaccination dose.

### Sex and dynamic response patterns predict antibody responses

As expected given the established enhanced serological effect of the AS03 adjuvant, antibody responses were less variable between individuals in the adjuvanted group than in the non-adjuvanted group^14^ (Figure 3E). Nonetheless, we asked whether any of the RPs might be associated with antibody titer response given that this homogenization was incomplete, with variability in titer responses even in the presence of adjuvant shown by a >30-fold range in MN titers at day 28. Using the elastic-net and cross-validation framework employed above, but here examining the adjuvanted and non-adjuvanted groups separately, we developed models to extract features that contributed significantly to the prediction of day 28 titers (Figures 3G and 3H; Figures S5A and S5B).

Fp02 was a predictive feature for both groups, but with opposing effects, associating with lower antibody titers in the adjuvanted group but with higher titers in the non-adjuvanted group (Figures 3G and 3H). While the predictive models were based on multiple parameters and each might exert only mild effects, comparable absolute effect sizes (α values) between the adjuvanted and the non-adjuvanted models support that Fp02 has opposing effects in the two groups. Fp02 captured primarily a transient increase, within hours of the first dose, in the frequency of activated CD8^+^ T cells and CD14^−^CD16^+^ monocytes (Figure 2E). This suggests that early activation and increases of these cells might promote antibody responses in the absence of adjuvant, but may detract from the adjuvant-induced processes that broadly increase antibody response quality and quantity.^17,31^

Sex was a consistent predictive feature for both adjuvanted and non-adjuvanted groups (Figures 3G and 3H). Females tended to have higher antibody responses but the association was more pronounced in the non-adjuvanted group, suggesting that the adjuvant may normalize some of the sex-dependent response differences. The influence of sex is consistent with evidence that females tend to mount more robust immune responses, but are more prone to reactogenicity, and our own earlier observation that females tend to have an elevated, more activated baseline immune state, positively associated with antibody responses to influenza and yellow fever vaccines as well as with plasmablast-associated flares in a subset of lupus patients.^24,32–34^

### Functional assessment of persistent cell states in an independent cohort

**Figure 4.**
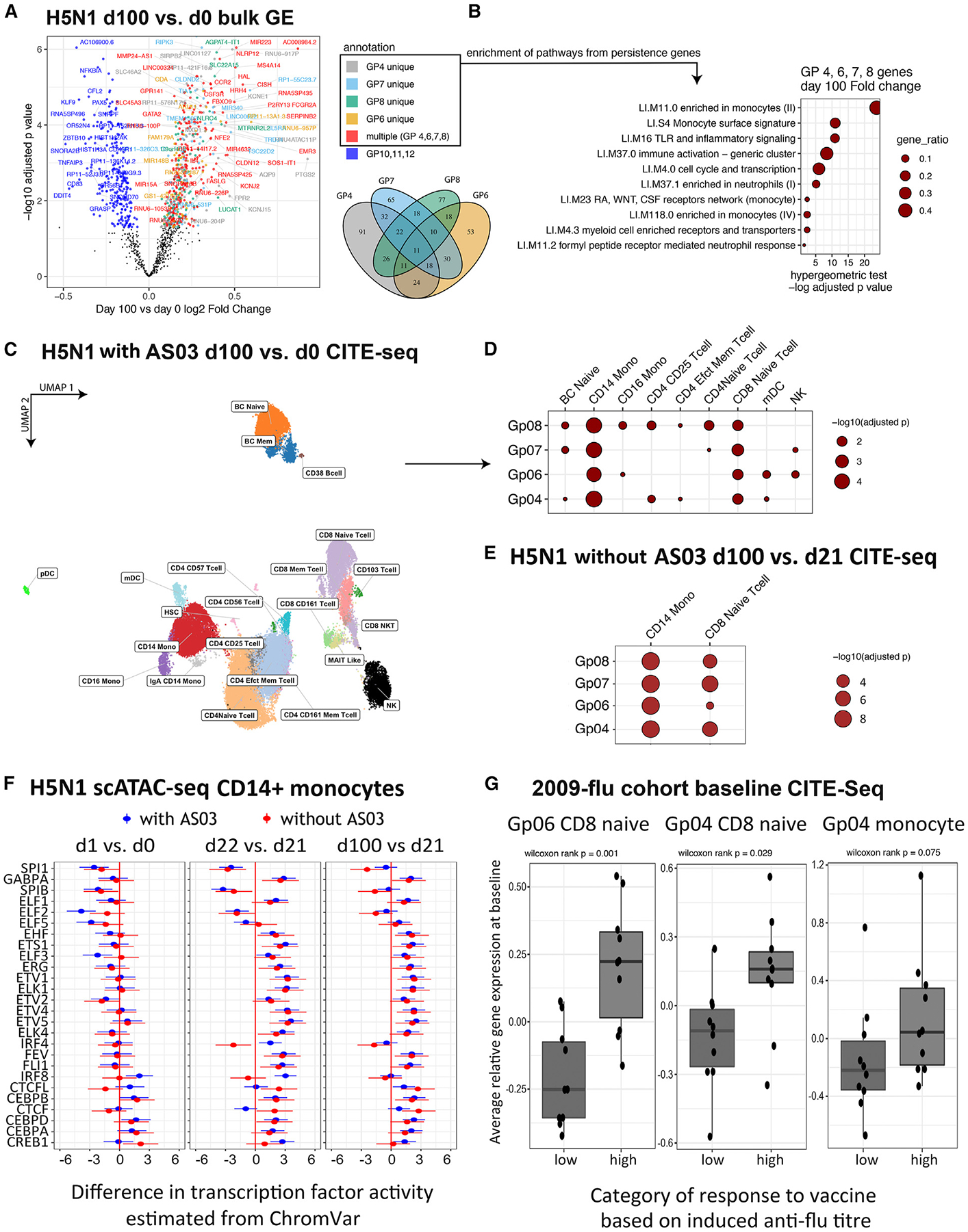
Multimodal single-cell analysis of persistent signatures and assessment in an independent vaccination study (A) Genes in the response patterns (RPs) that displayed persistent alteration at day 100 are shown in a volcano plot with day 100 versus day 0 gene expression fold changes (x axis) versus statistical significance (y axis). Genes in Gp04, 06, 07, 08, 10, 11, and 12 are included, and those with adjusted *p* < 0.01 are colored by their corresponding RP. Red indicates genes shared among Gp04, 06, 07, and 08 (RPs that displayed a persistently elevated trend by day 100); dark blue are genes in any of Gp10–12 (RPs that displayed a persistently depressed trend by day 100). The Venn diagram shows gene overlaps among the elevated RPs. (B) Hypergeometric test assessing enrichment of BTMs in the persistently elevated genes (those with *p* < 0.01 and positive fold change in A). Statistical significance of the hypergeometric enrichment is shown (x axis), with dot size denoting the gene ratio as defined by the clusterProfiler package. (C) For *n* = 6 adjuvanted subjects, single-cell CITE-seq analysis was performed for PBMC samples from days 0 and 100. A UMAP shows the major clusters identified, with protein expression reported in Figure S7A. (D) Enrichment analysis of persistent genes in specific cell types using the CITE-seq shown in (C). Within each cell population identified, “pseudo bulk” gene expression was used to rank all genes by t statistics from a mixed-effect linear model comparing day 100 versus baseline. This was followed by GSEA of the persistent genes with elevated expression from the indicated RPs. Here, persistent genes were determined from the day 100 versus day 0 comparison in (A), where adjusted *p* < 0.01 and fold change is positive—i.e., colored dots with positive fold changes in (A). (E) For *n* = 3 non-adjuvanted subjects, enrichment analyses show that the same RPs and cell types also demonstrate persistent effects in the absence of adjuvant. A further 13 subjects were analyzed by CITE-seq, of which 10 were adjuvanted and showed persistent effects of Gp04 and 06–08, similar to those that had been observed for the first 6 adjuvanted subjects studied in (D). Leading-edge genes from the enrichment of Gp04 and 06–08 in specific cell types in the 10 new adjuvanted subjects (Figure S7B) were then used for GSEA in the 3 non-adjuvanted subjects and for the selected cell populations now shown here. For this set of 13 individuals, CITE-seq assessed persistent effects at day 100 compared to day 21 rather than day 0, which was justified, as for Gp04 and 06–08 these patterns had previously been determined to decline to day 0 levels by the time of dose 2 on day 21 (Figure 2B). (F) scATAC-seq was generated for the 10 adjuvanted and 3 non-adjuvanted subjects described in (E) and identified transcription factors with similar differential accessibility both at the persistent time point (day 100 versus 21) and immediately after dose 2 (day 22 versus 21). Differential accessibility is shown for motifs enriched near the day 100 leading-edge genes, which had been observed for CD14 monocytes in (D), identified using centrimo with an e-value cutoff of 1e–10. The differential accessibility of these motifs was then determined by computing chromVAR accessibility scores and linear mixed-effect models. Shown is the scaled effect size and 95% confidence interval (1.96 × SE). (G) Assessing cell-type-specific persistent signatures for association with antibody response using baseline single-cell CITE-seq data from the 2009 flu vaccination cohort. Leading-edge genes from each of the persistent RPs (Gp04 and 06–08) enriched in classical monocytes and CD8^+^ naive-like T cells in (D) were tested. Boxplots compare average relative expression of these genes at baseline, between high and low antibody responders in the 2009 flu cohort, for the indicated gene patterns (Gp04 and 06) and cell types (monocytes or CD8^+^ naive-like cells). Additional results, including for dendritic cells, are in Figure S8C.

We observed that some of the RPs identified were persistently elevated (Gp04 and 06–08) or depressed (Gp10–12) even at day 100 after the first vaccination dose (Figures 1D and 2B). We tested whether these differences were specific to the adjuvanted subjects but found no such evidence. Instead, 142 genes from these persistently changed RPs were significantly different in gene expression at day 100 in both the adjuvanted and the non-adjuvanted group (false discovery rate [FDR] < 0.05). Similar BTM modules were enriched in both groups, and fold changes for these genes were also correlated between the adjuvanted and the non-adjuvanted group (Table S2; Figures S6A and S6B). We then pooled data from the adjuvanted and non-adjuvanted vaccinees together to derive genes from these RPs that were persistently elevated (non-black/blue dots in Figure 4A) or depressed (blue dots in Figure 4A). The elevated genes include those that are enriched in cells such as monocytes and neutrophils and in cellular activation and inflammation signatures, suggesting that even an antigen-dose-sparing vaccine without adjuvants can potentially leave a long-lasting mark in peripheral immune cells (Figure 4B). Since we do not have data from a placebo arm (e.g., saline only), it remains possible that factors other than vaccination were responsible for inducing this persistent immune state. However, subjects in our cohort were immunized over the course of 8 months, thus rendering seasonal variation and shared exposure to common pathogens less likely to account for the acquisition of this persistent immune state.

To dissect the cellular source of the persistent signatures, we initially applied CITE-seq to profile cell-surface proteins and transcriptomes simultaneously in 40,589 single peripheral blood mononuclear cells (PBMCs) from days 0 and 100 in six adjuvanted vaccinees (mean of 3,382 cells/sample). Clustering the single cells based on surface-protein expression revealed major lymphocyte and myeloid cell subsets (Figures 4C and S7A). We then computed gene expression differences between day 0 and day 100 within each cell cluster and assessed whether these cell-type-specific expression differences are enriched for the persistent signatures identified from bulk transcriptomics above. While most cell types were enriched for the persistently elevated genes in Gp08, only classical monocytes and a cluster of CD8^+^ naive-like T cells were enriched for all four persistently elevated RPs (Gp04 and 06–08), indicating that these two cell subsets had the highest relative enrichment of the persistence signal (Figure 4D).

To further investigate this persistent change in immune status and also examine whether it was seen in non-adjuvanted subjects at single-cell resolution, CITE-seq single-cell profiling was extended to an additional 13 subjects, including 10 adjuvanted subjects for whom we similarly observed persistent effects of Gp04 and 06–08, including in classical monocytes and CD8^+^ naive-like T cells (Figure S7B). Leading-edge genes from these enrichments were used for gene set enrichment analysis (GSEA) in the three non-adjuvanted subjects for which we had cells available from the required time points, which confirmed the above observation based on bulk data that, even in the absence of the AS03 adjuvant, Gp04 and 06–08 were persistently impacted and in the same cell populations of CD14^+^ monocytes and CD8^+^ naive T cells as we saw for the adjuvanted subjects (Figure 4E). These results together indicate that vaccination with or without the adjuvant can potentially set a new immune state within specific circulating cell populations that persists for at least 100 days (79 days after the boost).

To explore transcriptional regulatory origins of these persistent effects, we also generated scATAC-seq data on the same PBMC samples from the 13 subjects (10 adjuvanted and 3 non-adjuvanted) assessed by CITE-seq, including days 0 (before dose 1), 1, 21 (before dose 2), 22, and 100. The active regions (peaks 10 kb on either side of the gene body of leading-edge genes) in the persistent signature of Gp04 and 06–08 in CD14^+^ monocytes and CD8^+^ naive T cells were scanned for significant enrichment of binding motifs for TFs. These TFs were then assessed for differences in activities as reflected by chromVAR scores computed from the scATAC data; chromVAR reflects the extent to which a particular TF motif is found in accessible chromatin across the genome.^35,36^ We fit chromVAR scores in linear mixed-effect models comparing time points separately for adjuvanted and non-adjuvanted subjects. For both classical monocytes (Figure 4F) and CD8^+^ naive T cells (Figure S7C), multiple TFs showed similar altered chromVAR scores both at the persistent time point (day 100 versus 21) and immediately after dose 2 (day 22 versus 21), but not immediately after dose 1 (day 1 versus 0). This suggests a role for these TFs in shaping the persistent nature of the response specifically after dose 2 (although these could potentially be “primed” by dose 1 as well). Consistently, Gp04 and 06–08 signatures reverted to baseline at the mRNA level after dose 1, whereas a persistent response lasting out to day 100 was detected only after dose 2 (Figure 2B). These TF activity patterns were very similar between adjuvanted and non-adjuvanted individuals, in accord with the persistent transcriptional patterns being independent of adjuvant (Figure 4E). For classical monocytes (and to a lesser extent in the naive-like CD8^+^ T cells), the TFs with increased activity at both day 100 and day 22 compared to day 21 were dominated by members of the ETS (erythroblast transformation specific) family, including GABPA, EHF, ETS1, ERG, FEV, FLI1, and multiple ELK and ETV subfamily members. There is evidence for a broad role of ETS TFs in hematopoietic differentiation, including for myeloid cells.^37^ The specific detection of ETV1, 2, 4, and 5 may be particularly relevant in the control of monocyte functional states, as ETV subfamily members have been identified as major regulators of monocyte differentiation into macrophages or dendritic cells in response to inflammatory stimuli in mice and *in vitro* human studies.^38,39^ Also notable was the reduced activity of PU.1 (SPI1) in classical monocytes, which is also known to regulate monocyte differentiation.^40^ Thus, two doses of antigen-sparing vaccination can result in persistent cell-type-specific transcriptional states, even in the absence of adjuvant.

We next assessed whether similar persistent changes in blood transcriptional phenotype could be detected in an independent cohort of vaccinees who received the 2009–2010 pH1N1 + seasonal influenza vaccine without adjuvants. For this “2009-flu” cohort, PBMC transcriptomic data from the baseline and 70 days after vaccination have been previously reported.^29^ Differentially expressed genes from the persistently altered RPs after H5N1 vaccination above (Figure 4A) were found enriched for genes that altered in the same direction at day 70 compared to day 0 in this 2009-flu cohort (Fisher’s exact test *p* = 2.3 × 10^−8^) (Figure S8A). When ranking all genes by their changes in expression at day 70 compared to day 0 in the 2009-flu cohort, the highly ranked genes were significantly enriched for the persistently elevated RPs (Gp04 and 06–08; *p* < 1 × 10^−5^) (Figure S8B). These observations together suggest that the persistent changes observed 100 days after H5N1 vaccination (independent of the adjuvant) can also be detected more than 2 months after vaccination with a different vaccine (without adjuvant) in an independent cohort from a different year. Thus, persistent immune changes could be a consequence and perhaps even a common feature of vaccination.

To functionally assess the persistent signature, we asked whether this signature evaluated at baseline in an independent vaccination cohort may be associated with antibody responses following vaccination. We again used the 2009-flu cohort, comparing whether the persistent signature from the H5N1 analysis above differs between the high and the low antibody responders at baseline before vaccination. For this evaluation, we utilized the single-cell CITE-seq data we published previously involving the baseline assessment of 10 high- and 10 low-antibody responders from the 2009-flu cohort.^34^ Since monocytes and naive-like CD8^+^ T cells were enriched for genes from the persistently altered RPs (Gp04 and Gp06–08; Figure 4D), the leading-edge genes from these enrichments were assessed in the same cell types in the baseline CITE-seq dataset from the 2009-flu cohort. Strikingly, the individual RPs or their constituent genes combined all trended higher in CD8^+^ naive-like T cells in the high versus low responders from the 2009 cohort and were statistically significant for Gp04 and Gp06 (Figures 4G and S8C). The differences between the high and the low responders were milder but still tended to be elevated in classical monocytes, particularly for Gp04, and also for several of the RPs when assessed in myeloid dendritic cells (Figures 4G and S8C). Thus, two doses of antigen-sparing H5N1 vaccination (even without adjuvants) could induce a long-lasting transcriptional state in classical monocytes and CD8^+^ naive-like T cells associated with elevated antibody responses in an independent seasonal influenza vaccination cohort.

## DISCUSSION

We present here a comparative analysis of human responses to H5N1 vaccination with or without AS03, using a novel unsupervised dynamic RP discovery and scoring approach that enabled the linking of individual response dynamics to outcomes such as antibody titers. This approach is generally applicable and may prove particularly useful as our ability to generate dense, time-resolved datasets in human cohorts continues to advance. Our analyses uncovered adjuvant-specific signatures and their dynamics, including early responses that differ between the first and the second dose of vaccination, and RPs predictive of serological responses. Unexpectedly, we observed that independent of the adjuvant, two doses of H5N1 vaccination altered the transcriptional state of monocytes and CD8^+^ naive-like T cells out to at least 100 days after the first dose of vaccination. Such establishment of a new immune baseline state has the potential to impact future responses, as supported by our observation that these cell-type-specific baseline transcriptional signatures are associated with higher antibody responses to influenza vaccination in an independent cohort.

The AS03-induced signatures we describe showed overlap with previous reports.^9,14^ An earlier analysis of H5N1 vaccination responses using sorted peripheral blood cell populations also identified IFN signaling in dendritic cells, monocytes, and neutrophils.^9^ Similarly, in AS03-adjuvanted hepatitis B vaccination, transcriptional modules similar to aspects of the innate and IFN responses we observed were identified; these tended to peak 1 day after vaccination, with a stronger response after the second dose, along with a subsequent transition to adaptive responses.^14^ There were also some differences among these studies. For example, neither our work nor that of Howard et al.^9^ detected a prominent decrease in NK cell modules as reported by de Mot et al.,^14^ suggesting that this signal may be vaccine or antigen dependent or partly due to differences in prior exposure to influenza compared to hepatitis vaccine antigens.

Frequent sampling within the first 24 h after both vaccine doses was a unique feature of our study that helped pinpoint the timing and relative intensity of the early inflammatory and IFN responses, as well as other early response features such as monocyte activation after both vaccine doses. It also helped to reveal the response differences between the first and the second vaccine dose. Intriguingly, Gp01, which reflected monocyte activation, was more elevated after the initial than the second dose, perhaps due to a phenomenon similar to endotoxin tolerance whereby repeated inflammatory activation of monocytes can result in attenuated future responses.^41–43^ However, here, the two doses were given 21 days apart, and thus any such attenuation might be due to changes in progenitor cells or cell-extrinsic factors given that circulating monocytes have a half-life of just a few days.^44^ Other RPs that comprised the adjuvant signature also peaked within 1 day following either vaccination dose, but some showed increased intensity and peaked earlier after the second dose (e.g., Gp02). This may reflect an increased propensity of certain cells to respond after the first dose, although the mechanism could also be cell extrinsic, e.g., the cells could have been modulated by sustained cytokine responses from the first dose. Together, these features of the early (innate) response may determine how AS03 enhances subsequent adaptive responses, although further work is needed to dissect the mechanistic links between the RPs we uncovered and the beneficial effects of AS03, such as the improved breadth of immunity to drifted virus strains.^17,20,21,23^

Our unsupervised pattern discovery revealed persistently altered immune profiles after H5N1 vaccination, even with the antigen-sparing vaccine alone in the absence of the AS03 adjuvant. This persistent signal comprises transcriptional changes in classical monocytes and CD8^+^ naive-like T cells potentially shaped by the ETS family of TFs. Supporting the idea that this persistent immune state may have functional relevance, these signatures evaluated at baseline (before vaccination) are associated with better antibody responses to seasonal influenza vaccination in an independent cohort. These results together suggest that vaccination alone, even with a dose-sparing formulation, has the capacity to alter immune states months post vaccination and with the potential to impact responses to future vaccinations. Interestingly, this persistence signature arose only after two doses of vaccination with or without the adjuvant, as supported by our transcriptional (bulk and single cell) and chromatin-accessibility data (single cell). Furthermore, the changes that persisted to day 100 were largely already induced by day 22, 1 day following the second vaccination. By contrast, this signature had reverted back to baseline (day 0) by day 21, immediately before the second vaccine dose was administered. These data suggest that the first vaccine dose might have “primed” cells such as the long-lived progenitors of CD14^+^ classical monocytes (as those monocytes themselves tend to have a half-life of just a few days) in such a way that it takes a second dose to induce the longer-lasting effects we observed. However, the mechanisms behind this phenomenon remain to be determined.

Our observations are consistent with accumulating evidence that baseline, pre-vaccination states can be correlates and potential determinants of vaccination outcomes.^29,34,45–50^ Examples include healthy individuals who possess a “naturally adjuvanted” baseline immune state,^51^ so named because at baseline it resembled the inflammatory response induced by AS03 within 24 h of vaccination.^52^ In addition, we have previously reported a broadly activated baseline immune state that predicts antibody response to seasonal influenza and also to yellow fever vaccination.^34^ These baseline immune profiles appear distinct from the cell-type-specific persistent signatures described here. However, together, they begin to provide support for how baseline immune states may be manipulated *in vivo* to modify subsequent responses in humans, including enhancing responsiveness to vaccination independent of vaccine antigen specificity,^50^ as has been proposed earlier to exploit heterologous effects of vaccination.^53^ Nonetheless, further work is needed to assess whether these signatures are merely correlative or indeed point to interventional targets for safe modulation without increasing the risk for autoimmunity and other inflammatory conditions.

Our observations of altered timing and magnitude in early responses to the second dose of AS03-adjuvanted vaccination compared to the first dose, and long-lasting change in immune states post vaccination, are broadly consistent with the concept and phenomenon of trained immunity.^54^ In contrast to antigen-specific memory, trained immunity posits that prior inflammatory encounters by cells and organisms can induce long-lasting, antigen-non-specific innate memory, which in turn can influence responses to future perturbations. As highlighted above, known trained immunity mechanisms, including chromatin modifications in myeloid cells and their progenitors in the bone marrow, may underlie some of our observations.^54–57^ An earlier study^58^ showed that adjuvanted AS03-H5N1 vaccination can result in transcriptional and chromatin accessibility changes in blood monocytes that persist for 21–28 days after boost vaccination. The AS03-specific effects include enhanced accessibility of chromatin loci enriched for IRF TF binding motifs, increased interferon-stimulated gene expression, and elevated resistance to viral infections in blood monocytes. While our findings are conceptually similar to those of the Wimmers et al. study and to how BCG vaccination can induce changes in PBMC states 28 days post vaccination, with potential lowering of viremia after subsequent live yellow fever vaccination (attenuated infection),^55^ here, the phenomena we observed and the underlying mechanisms are likely distinct: (1) the persistent changes we detected could be attributed to early responses to vaccination with or without the adjuvant (1 day after both the first and the second vaccination), (2) they persisted to at least 79 days after the second vaccination, and (3) the chromatin accessibility signatures and TFs implicated are distinct from those (e.g., AP-1 and IRF) reported in these earlier studies 21–28 days after vaccination.^58^ Our results thus revealed how even a dose-sparing, non-live vaccine without adjuvant can induce long-term immune changes with potential positive functional impacts on future responses.

In addition to classical monocytes, single-cell analysis of our persistent signature pointed to CD8^+^ naive-like T cells. Even though these are lymphocytes often associated with adaptive immunity, the transcriptional signatures we detected are unlikely to mark antigen specificity, as such cells are generally low in frequency in circulation, particularly prior to or 79 days after vaccination, and the level of influenza-specific cellular immunity in circulating B and CD4^+^ T cells before vaccination is also not known to correlate with responses.^29,59,60^ Interestingly, mechanistic studies of the mode of action for adjuvant AS01 have also implicated non-antigen-specific CD8^+^ T cell responses constituting a major source of early IFN-γ production in draining lymph nodes^61^ and, similarly, in our earlier study of influenza vaccination responses, in subjects fully recovered from prior mild, non-hospitalized COVID-19.^49^ Together, the results of our work raise the possibility that cell-type-specific baseline immune states associated with enhanced vaccination responses can potentially be trained by vaccines themselves in an antigen-agnostic manner.

### Limitations of the study

Since we do not have data from a placebo arm (e.g., saline only), it remains possible that factors other than vaccination with antigen were responsible for inducing this persistent immune state. However, subjects in our cohort were immunized over the course of 8 months, thus rendering seasonal variation and shared exposure to common pathogens less likely to completely account for the acquisition of this immune state. Indeed, similar persistent changes were detected 70 days after vaccination in an independent 2009–2010 pH1N1 + seasonal influenza vaccine cohort with a wide range of participant ages. The TF binding and activity analyses were based on chromatin accessibility data only; thus, they should be interpreted with caution. However, our observations and conclusions on persistence signatures are supported by multiple lines of evidence, including both bulk and single-cell transcriptomic data, and functional assessment of the signature in an independent vaccination cohort in which the persistent signature was recapitulated by the baseline status of high-antibody responders, thus suggesting that the persistent signature we identified has functional relevance. However, it remains to be determined how long such persistent effects last and whether other vaccines exert similar long-term impacts on the human immune system.

## RESOURCE AVAILABILITY

### Lead contact

Requests for further information and resources and reagents should be directed to and will be fulfilled by the lead contact, John Tsang (john.tsang@yale.edu).

### Materials availability

This study did not generate new unique reagents.

### Data and code availability

Unprocessed data are available for microarray, flow cytometry, Luminex, and titer assays in Immport (study no. SDY1252) and for CITE-seq and ATAC-seq in Zenodo; data are publicly available as of the date of publication. All original code with a Singularity container and workflow document (Data S1) is made available in the same Zenodo repository. The DOI is listed in the key resources table. Any additional information required to reanalyze the data reported in this paper is available from the lead contact upon request. Note that data from three subjects are not included in the datasets made available due to the type of data reuse consent given by these three individuals. Data are provided for all assays with these three subjects removed and for CITE-seq after demultiplexing (of pooled subjects) and removal of these three individuals.

## STAR★METHODS

### EXPERIMENTAL MODEL AND STUDY PARTICIPANT DETAILS

#### Study participants

This study was approved by the IRB of the NHLBI (12-H-0103) with written informed consent obtained from eligible healthy volunteers prior to any study procedure. 42 subjects were studied including representatives of multiple races (60% Caucasian, 24% African American), both sexes (60% female), and with ages ranging from 22–44 years (Table S1). Exclusion criteria included receipt of AS03-adjuvanted vaccine at any time in the past, or seasonal influenza vaccine within the previous 3 months.

#### Vaccine characteristics

Subjects were vaccinated with antigen either with or without AS03 (GSK), which is an Adjuvant System containing DL-α-tocopherol and squalene in an oil-in-water emulsion^19^. The vaccine contained inactivated split-virion influenza with 3.75 mcg of HA antigen from the clade 2.1 strain A/lndonesia/05/2005, which has been approved by the US Food and Drug administration and is currently in the US National stockpile for preparedness against pandemic influenza^16,30^. The observed vaccine response rates, HAI titers, time of peak response, and response durabilities are shown for the 42 subjects in this study (Figures S9A–S9D).

### METHOD DETAILS

#### Sample collection

Baseline blood samples were drawn from fasting individuals and were processed within 30 minutes of drawing. Serial timepoints of blood samples were collected on the following schedule: before vaccination; at 0 hours, 2 hours, 4 hours, 12 hours, 1 day, and 7 days for each of the primary (d0) and boost (d21) vaccinations; and at day 42 and day 100 post-vaccination (Figure 1A). For each of the 24hr periods including 5 sampling points, subjects were hospitalized with an indwelling venous catheter placed in the forearm. Peripheral blood mononuclear cells (PBMC) were isolated using Leucosep tubes (Greiner bio-one, Germany) and Ficoll-Paque Plus (GE Health-care, Sweden) density gradient centrifugation. An aliquot of fresh PBMC was lysed in QIAzol (Qiagen, Hilden, Germany) and immediately kept at −80°C freezer for later RNA isolation. The remaining PBMC were cryopreserved in 90% heat inactivated fetal bovine serum (HI-FBS; Gibco, Grand Island, NY) and 10% dimethyl sulfoxide (Sigma-Aldrich, St. Louis, MO). Serum was obtained by centrifugation of peripheral blood collected in serum separating tubes (SST) (Beckton Dickinson, San Jose, CA) according to manufacturer’s protocol. PMBC and serum samples were stored in liquid nitrogen until use. The assays that were performed on samples from each time point are detailed in Figure 1A, and Table S1.^65^ Before analyses, all samples were batched by an independent investigator to randomize age, gender, race and immunization status. For all assays samples were run blinded to immunization status.

#### H5N1 antibody titer determination

Vaccine induced antibody titers were assessed by both virus neutralization and hemagglutination inhibition assays. Microneutralization (MN) titers assessed viral-neutralizing activity using MDCK cells based on the methods of the pandemic influenza reference laboratories of the Centers for Disease Control and Prevention (CDC), with minor modifications provided in an updated SOP issued by the CDC. MN titers were measured against H5N1 vaccine strains of A/Vietnam/1194/2004 (clade 1), A/Indonesia/5/2005 (clade 2.1), A/Turkey/15/2006 (clade 2.2), A/Egypt/3072/2010 (clade 2.2), and A/Anhui/1/2005 (clade 2.3.4) as well as the H1N1pdm09 (A/California/7/2009). Sera were tested at an initial dilution of 1:20, and those that were negative (<1:20) were assigned a titer of 10. All sera were tested in triplicate, and the geometric mean value was used for analysis. The titers represent the reciprocal of the highest dilution of serum which completely suppressed virus replication.

Hemagglutination inhibition (HAI) titers were determined as previously described^66^, after treating sera with sialidase (RDE II; Vibrio cholera Ogawa type 558 filtrate; Denka- Seiken, Tokyo, Japan). Briefly, 3 volumes of sialidase were added to 1 volume of serum and the combination incubated at 37C for 18 hours. 3 volumes of 2.5% sodium citrate were then added and the mixture incubated at 56C for 30 minutes before 3 volumes of PBS were added for a dilution of 1:10 for each sample. 25μL of a 2-fold dilution in PBS of the RDE-treated sera were placed in a 96 V-well microtiter plate, and a further 25μL added containing 4 HA units of the influenza strain H5N1-A/Vietnam/1203/2004. After incubation at room temperature for 30 minutes, 50 μL of a 0.5% suspension of red blood cells was added, followed by incubation at 4C until red blood cells in the PBS control sample formed a button. HAI titer was defined as the reciprocal of the highest dilution of serum that inhibited red blood cell hemagglutination by influenza virus.

#### Bulk transcriptomic data generation

For transcriptomic analysis PBMC were stored in QIAzol as described above. For PBMC, total RNA was isolated and purified with the miRNeasy kit (Qiagen). RNA quality and quantity were estimated using Nanodrop (Thermo Scientific, Wilmington, DE) and Agilent 2100 Bioanalyzers (Agilent Technologies, Palo Alto, CA). RNA was amplified from 200 ng of total RNA using the Ambion WT Expression Kit (Thermo Scientific). Fragmented single-stranded sense cDNA were terminally labeled and hybridized to the Affymetrix Human Gene ST v2.1 Array Plate (Affymetrix, Santa Clara, CA) with 53,617 probesets for 40,716 RefSeq coding and noncoding transcripts and 11,086 lncRNA transcripts. The sample order was spatially randomized in the array plate. The arrays were then washed, stained and finally scanned on a GeneTitan MC instrument (Affymetrix) controlled by the AGCC GeneTitan Control software.

The raw intensities of gene probesets in the Affymetrix CEL files were processed using Affymetrix Power Tools (http://www.affymetrix.com/support/developer/powertools/index.affx). The “apt-probeset-summarize” script from the Affymetrix Power Tools was used for background correction and RMA with sketch quantile normalization. The resulting probeset level intensities were extracted and converted to an “Expression Set” object using Bioconductor’s Biobase package in R programming language. The total number of samples was 750 for PBMC, including reference and duplicated samples. The reference and duplicated samples (except duplicated baseline samples) were removed and not included in further analysis. In addition, probesets associated with multiple genes were removed. When a gene was associated with multiple probesets, we computed the principal components of these probesets across all arrays and only use the probeset that correlated the most to the first principle component. After these filtering steps, 29365 genes were left. The Bioconductor package “arrayQualityMetrics” was used to assess quality of the microarray data.

#### Flow cytometric immunophenotyping

After thawing, 10^7^ cells were washed in PBS FACS buffer (1X Phosphate-Buffered Saline, 0.5% Fetal Calf Serum, 0.5% Normal Mouse Serum and 0.02% NaN_3_) and incubated with LIVE/DEAD Fixable Dye (a viability dye), which was used to exclude dead cells from analysis (Life Technologies, Carlsbad, CA). Cells were washed and re-suspended in 100ul of FACS buffer and incubated with fluorochrome-conjugated antibodies in lyoplates developed as part of the Human Immunophenotyping Consortium^67,68^. The staining panels used were five parallel 10 color panels, with a total of 30 unique markers, designed to characterize T cells, regulatory T cells, helper T cells, B cells and DC/monocyte/NK lineages (Table S3). After incubation with antibodies for 30 minutes, cells were washed two times with FACS buffer, and fixed in 1% paraformaldehyde. Cells were then acquired on a LSR-Fortessa flow cytometer equipped with 355, 405, 488, 532 and 633nm laser lines using DIVA^™^ 8 software (Becton Dickinson). For each sample, 250,000 cells were acquired. The resulting data were then analyzed with FlowJo^™^ software version 9.7.6 (Treestar, San Carlos, CA). Compensation performed with unstained cells and compensation beads (Becton Dickinson), was used during acquisition of the specimen to ensure recording of enough events for the populations of interest. Subsequently a final compensation matrix was calculated using FlowJo during post-acquisition analysis. 84 immune cell populations were characterized, according to the marker definitions listed in Table S4. Each population was expressed as the percent of parent gate, with counts and percent of total also reported. For one subject, H5N1–010, cytometry data was not obtained for the 4 and 12 hr timepoints from d21.

#### Serum protein analysis

For all time points serum was analyzed using Luminex to measure 76 cytokines, chemokines and acute phase proteins using 5 human cytokine multiplexing panels (Bio-Rad, Hercules, CA, USA) (Table S5). All assays were performed according to Bio-Rad kit protocols. Median fluorescence intensities were measured on a Luminex 200 instrument (Luminex, Bio-Rad, Hercules, CA, USA). Standard curves for each cytokine were generated using the premixed lyophilized standards provided in the kits. Concentrations in samples were determined from standard curves using a 5 point-regression to transform mean fluorescence intensities into concentrations using Bio-Plex Manager software version 6.1. Each sample was run in duplicate and the average of duplicates was used as the measured concentration. Concentrations are expressed in pg/mL unless stated otherwise (for acute phase proteins). To facilitate inter-individual comparison, results were analyzed as fold change to day 0 (0h).

#### Single cell sequencing-based analyses

CITE-seq data was generated using PBMC samples from H5N1 vaccine recipients in two groups. Initially for 6 adjuvanted subjects at days 0 and 100, then subsequently for a further 13 subjects (10 adjuvanted, 3 non-adjuvanted) at days 0,1,21,22 and 100. CITE-seq was performed using the same experimental protocol, antibody panel and demultiplexing procedure as previously reported^34^. Reads from mRNA were aligned using Cell Ranger and antibody derived tags (ADT) were aligned using CITE-seq-count^69^.

Single cell ATAC-seq data was generated for the same set of PBMC samples analysed by CITE-seq, from 13 subjects at days 0,1,21,22 and 100. In each run the cells from six or seven subjects at one time point were combined together as one pool and processed using Chromium Next GEM Single Cell ATAC Reagent Kits v1.1 (10X Genomics). Briefly, nuclei were isolated according to the manufacturer’s protocol, transposed and then super-loaded at about 30,000 nuclei per lane into 10X Chip H with two lanes used for each pool. Libraries were constructed following the manufacturer’s instructions, pooled into one tube, and sequenced on a NovaSeq 6000 with S4 Reagent Kit (Illumina).

#### Reference samples, batching control, and data QC

To minimize intra-individual variations, blood samples from different time points from the same subject were processed at the same time. To control for batch variation, healthy donor reference blood samples obtained by leukapheresis from the NIH Department of Transfusion Medicine, were simultaneously processed with the experimental samples according to the assay performed. For transcriptomics, RNA was isolated in parallel from a reference sample and from experimental samples, and the reference sample included in each array. For flow analysis, one reference sample was processed in each lyoplate (CHI-010), on each day staining was performed, to assess inter-plate variation. For Luminex, serum samples were run in 3 batches, with baseline d0 (0h) samples run in each batch along with a reference serum (CHI-001) to allow normalization among batches. For the Somascan Assay, inter-run normalization was performed using a CHI specific bridge sample (QC_CHI1), in addition to SomaLogic provided calibrators, as previously described^70^.

During RNA data processing quality control, one sample was detected as a clear outlier by PCA and was removed from the dataset. PCA was performed using only chromosome Y linked genes from the corresponding blood transcription module, to test that subjects’ gender match their sex-specific transcription profile. Two samples were detected to have swapped labels in both the PBMC and PAXgene datasets, and were corrected. Four other samples were also detected to have a wrong label and were removed from the dataset. Two of those samples were day 0 samples of subject H5N1–010, thus all timepoints from this subject were removed when calculating fold changes for pattern discovery. We introduced samples from this subject back later (except day 0) for adjuvant status prediction. For PBMC samples, probesets with very low variation (with an inter-quantile range of less than 0.15) and without annotated gene symbols (based on Affymetrix’s latest annotations downloaded from their website) were filtered out.

### QUANTIFICATION AND STATISTICAL ANALYSIS

#### Pattern discovery for blood transcriptomic data

##### Samples and genes selection

PBMC samples were used to discover longitudinal patterns in gene expression data. The fold change of genes at each time point were computed by subtracting their mean gene intensities from the mean gene intensities at the zeroth hour of day zero. Genes with a log fold change of more than 1 in a minimum of 30% of the samples at any time point were selected, leading to 202 genes and 8282 (41 subjects × 202 genes) subject-gene combinations.

##### Clustering and pattern discovery

Starting from a matrix of longitudinal profiles (14 time points; rows) by subject-gene combinations (columns) we calculated a matrix of Pearson correlation coefficients between all subject-gene combinations. This correlation matrix was used as a dissimilarity matrix for divisive clustering with the DIANA algorithm implemented in the “cluster” R package^71^. Next, we applied a series of cuts at different heights to the clustering tree to search for the most stable clusters. Stable clusters were defined as those with the largest difference between the heights that divide a cluster, and that possessed more than 200 and less than 2000 nodes (subject-gene combinations) corresponding to a minimum of 10% of the subjects. Nodes that didn’t fall into any of these clusters were removed. Clusters with more than half of the nodes having a negative silhouette width were also removed. This tree cutting algorithm resulted in 14 clusters with distinguishable longitudinal patterns. From each cluster/pattern we removed nodes with less than 25% of positive correlations and with genes having only one subject within that pattern. The pattern profile was calculated as mean of fold change of nodes within the cluster.

##### Pattern gene signatures

Having established pattern profiles, we developed a procedure to extend the number of genes associated with each pattern. We applied relaxed criteria for gene selection with a fold change of more than 0.5 in a minimum of 20% of the samples. These relaxed criteria lead to a selection of 6672 genes that includes 202 genes previously found based on the more stringent criteria. Then for every subject-gene combination we calculated the correlation between its profile and each pattern profile (Figure S1A). A gene was assigned to a pattern if correlation for at least 30% of subjects was positive with a p-value of less than 0.01. Then each pattern’s gene by subject matrix of correlation coefficients was clustered with k-mean (K=2)^72^ and genes in a cluster with smaller average correlation were removed (Figures S1B and S1C). Two patterns with lowest quality metrics—Gp09 and Gp10—were excluded from elastic net modeling to predict titer responses (Figure S1C).

##### Enrichment of blood transcription modules in pattern signature genes

The enrichment p-values were computed for Li^26^ modules using a hyper-geometric test with pattern signature genes as a foreground and previously selected 6672 genes as a background. The p-values were adjusted using the Benjamini and Hochberg method^73^. R package “tmod” was used for all enrichment calculations.

##### Pattern scores for individual subjects

First, the average z-scores were computed for all the samples using pattern signature genes. A correlation of sample scores for the subject against the pattern profile defined the subjects score for a given pattern. To compute pattern scores for previously removed subject H5N1–010 due to missing baseline, the fold change was calculated using d21(0h) as the baseline.

#### Pattern discovery for cell population frequencies

Cell subpopulation data were reported as a percentage of parent frequencies by sequential manual gating of 10-color flow cytometry stains (T cell, T helper, T reg, B cell, and DC/monocyte/NK). A total of 84 cell subpopulations were thus initially considered for all 42 subjects across 11 timepoints. For each cell subpopulation and subject, the normalized longitudinal profile was obtained by subtracting the baseline and dividing by the median across all timepoints. A cell subpopulation was kept in the analysis if, for at least one time point, >30% of the cohort had a coherent change (i.e. increase or decrease) of the normalized frequencies above 50%. With these filtering criteria, 15 flow IDs representing 13 cell subpopulations (see Figure 2E) were used in the subsequent clustering analysis. From all cell subpopulation-subject combinations taken pairwise, a similarity matrix was built based on the distance d=sqrt(2(1-r)), where r is Pearson’s correlation; this definition of distance satisfies ultrametric properties^74^. Hierarchical clustering was performed on the similarity matrix using the UPGMA agglomeration method implemented in the “hclust” function from the R package “stats”. The dendrogram produced from the hierarchical clustering was then cut sequentially using the “cutreeHybrid” function from the R package “dynamicTreeCut”(^75^). We performed a grid search over two parameters affecting this tree-cutting function, namely: deepSplit (an integer in the 0–4 range that provides control over cluster splitting sensitivity) and the minimum cluster size (which we chose as either 5% or 10% of the number of cell subpopulation/subject combinations). For each parameter combination in the grid search, the “cluster.stats” function from the R package “fpc” was used to determine cluster quality measures (used as objective functions for the clustering procedure) such as the mean intra- vs inter-cluster distance, the average silhouette width, the Pearson version of Hubert’s gamma coefficient, the Dunn index, and the Calinski and Harabasz index. From the analysis of these cluster quality measures, we chose the parameter combination (deepSplit=0, minimum cluster size=5%), which led to 8 longitudinal patterns.

Up to this point, clustering was performed on all cell subpopulation-subject combinations; the next set of steps was aimed at disentangling this information to 1) assign patterns to cell populations, and 2) obtain subject scores associated with each pattern. To proceed, we first built a cell subpopulation × subject matrix, where each entry was the pattern label assigned to that particular combination; this label was missing for some entries that were not assigned to any patterns. For each pattern label, we converted this matrix to binary according to the logical “cell subpopulation-subject combination was assigned to the pattern”; given any two cell subpopulations, we computed their similarity as the Hamming distance across subjects in the binary matrix. On this cell subpopulation similarity matrix, we computed K-means clustering with K=2, which partitions cell subpopulations in two groups. Looking back at the binary matrix for each group, we computed the fraction of 1’s; the group with the largest fraction was assigned to the pattern. By repeating this procedure over all patterns, we were able to effectively associate patterns with specific sets of cell subpopulations. Subject scores associated with each pattern were then obtained as the Pearson’s correlation between a subject’s mean longitudinal profile (averaged over cell subpopulations associated with the given pattern) and the pattern’s profile (Figure S2A,B).

#### Regularized linear regression modeling with elastic net

Linear regression modeling was performed using our “eNetXplorer” R package that was designed as a toolkit for the quantitative exploration of elastic net families for generalized linear models^76^ (Figure S3A). The elastic net for generalized linear models is a popular regression and feature selection method, particularly useful when the number of features is substantially greater than the sample size and when there are many correlated predictor features. The mixing parameter α (alpha) generates a family of models with increasing shrinkage effects from ridge (α=0) to lasso (α=1); for each of them, an ensemble of solutions depends on the penalty parameter λ (lamda), which is chosen by cross-validation. Using eNetXplorer, statistical significance was assigned to each model by comparison to a set of null models generated by random permutations of the sample labels; analogous permutation procedures were used to assess significance for the contribution of individual features to prediction. Criteria for model and feature selection are as follows: 1) a family of elastic net models is generated using α=0, 0.1, 0.2, … 1; 2) the most significant model is selected (i.e., α that maximizes −log10(p-value of model vs null); 3) features with mean nonzero coefficient p-value<0.20 are selected. To compare blinded and unblinded models, e.g. a model using subjects predicted to be adjuvanted (POS) during the blinded phase compared to a model using the actual adjuvanted (ADJ) subjects after unblinding, a common value of α was chosen based on significance from both blinded and unblinded distributions.

#### Validation of adjuvant signature using microarray data from Emory University

##### Data processing

We received the gene expression data as a matrix of RMA summarized intensities of Affymetrix probesets by all samples. The same procedure as above was used to convert the probesets to genes. The probeset annotation was downloaded from GEO’s GPL13158 platform.

##### Two-peak response pattern score calculation

As for our dataset we computed average z scores for all samples for Gp01, Gp02 and Gp03 response patterns. The Emory dataset had different time points, thus we estimated the pattern scores for each subject not as correlation with pattern profile, but as an average between fold change from d0 (0h) to d1 and fold change from d21 (0h) to d22. Prediction of adjuvant status in the subjects from Emory was performed by combining scores from Gp1, Gp2 and Gp3 response patterns to either cluster subjects, or to rank subjects followed by categorisation based on the number of subjects that received adjuvant (Figure S4B).

#### Exploration of persistent effects at day 100 after H5N1 vaccination

##### Microarray analysis

For gene expression RPs showing a persistent signal: Gp04, 06, 07, 08 elevated at day 100; and Gp10, 11, 12 decreased on day 100; all genes were tested for differential expression day 100 post vaccination compared to baseline using a linear model with a random intercept for each donor on normalized microarray data, using the dream implementation of lme4 and limma^63,64,77^. Genes with adjusted p values < 0.01 were then tested collectively (the union of pattern genes) or separately for each elevated persistence pattern for enrichment within the Li Blood Transcriptional Modules using a hypergeometric test implemented with the ClusterProfiler package^78^.

##### CITE-seq analysis of persistent signatures

CITE-seq data was generated using PBMC samples from H5N1 vaccine recipients in two groups. Initially for 6 adjuvanted subjects at days 0 and 100, then subsequently for a further 13 subjects (10 adjuvanted, 3 non-adjuvanted) at days 0,1,21,22 and 100. The first set of 6 individuals were used for single cell analysis of persistence signatures. Single cell mRNA from day 0 and day 100 H5N1 vaccinees were normalized and scaled by each cell’s library size to log transcripts per 10,000, and CITE-seq surface protein data was normalized using dsb implementing background correction with empty droplets and per-cell normalization factors derived from isotype controls and background protein levels in each cell^79^. Cells were clustered with a distance matrix defined by *dsb* normalized protein levels using Seurat and resulting clusters were annotated and purified based on canonical immune cell protein expression^62^. Gene expression fold change between baseline and day 100 within each protein defined cluster was assessed using dream with a random intercept for each donor^77^. Genes ranked by t-statistic were assessed for enrichment of gene pattern genes that had adjusted p < 0.01 and positive log fold change from the microarray day 100 versus baseline analysis (see above) using the fast gene set enrichment package^80^. The second set of 13 individuals were used to assess persistent signatures at single cell resolution in non-adjuvanted individuals. Analyses were repeated as above for the adjuvanted subjects, before leading edge genes from the enrichment of Gp4,6–8 in specific cell types in the 10 new adjuvanted subjects were used for GSEA in corresponding cell populations for the 3 non-adjuvanted subjects.

##### ATAC-seq analysis of persistent signatures

Single cell ATAC-seq data was generated for the same set of PBMC samples analysed by CITE-seq, from 13 subjects at days 0,1,21,22 and 100. For data processing demultiplexing of samples was performed using demuxlet. Quality control and clustering of cells was performed with ArchR with the default parameter settings. Briefly, low quality cells were filtered. Batch correction was performed to remove subject or batch variation in the LSI embedding with Harmony. Cells were clustered using the batch corrected LSI embedding of tile matrix. Cells were then mapped to d100/d0 RNAseq reference using the canonical correlation method of Seurat, and clusters enriched in CD8 Naïve and CD14 Mono predicted cells were assessed. CD14 monocytes were filtered for those within enriched clusters and predicted as that cell type. CD8 naïve cells utilized all cells predicted to be CD8 naïve cells by the label transfer method. Peaks were called within cell clusters using Macs2. For identification of differentially accessible motifs, motif activity scores were assigned to each single cell using the chromVAR implementation within ArchR. Pseudobulk libraries for each sample (Subject × timepoint combination) were created by taking the mean chromVAR score across all cells within the cells annotated naïve CD8 and CD14 monocytes. Linear mixed effect models were then fit for each motif using the dream() function from the variancePartition R package, with the following formula: motif ~ timepoint + FRIP_Mean + X + (1|batch) + (1|Subject), where X is a cubic spline basis matrix with 3 degrees of freedom to model the effect of library size, and FRIP_mean is the mean frip score for all cells in the pseudobulk library. Motifs that became more or less accessible over the timecourse were then identified by contrasting d1 to d0, and d100 and d22 to d21. Enrichment of transcription factor motifs in regions near leading edge were then assessed. Transcription factor motifs enriched near the d100 leading edge genes from the CD8 naïve and CD14 monocytes were identified by taking the intersection of all peaks from each cell type respectively with the regions 10KB upstream and downstream of all leading edge genes. Motif enrichment was then determined using the Centrimo enrichment test from the MEME suite to identify motifs enriched towards the center of the peaks near the LE genes.

#### Analysis of persistent effects in the context of 2009-flu vaccination

##### Bulk expression analysis at day 70

RNA expression data has previously been reported including for days 0 and 70 after vaccination for 2009–2010 pH1N1+seasonal influenza. This was under clinical trial NCT01191853 in which subjects received both the 2009 Fluvirin seasonal influenza (Novartis) and H1N1 pandemic (Sanofi-Aventis) vaccines^29^. Differentially expressed genes on day 70 compared to baseline were assessed using the dream method with the same donor random intercept model used for the H5N1 analysis comparing day 100 to baseline^77^. Gene patterns which had persistent effects on day 100 following H5N1 vaccination were then assessed in this independent 2009-flu vaccination cohort on day 70 using two methods. First, for the RPs that were persistently altered after H5N1 vaccination (Gp 04, 06–08, 10–12), all genes which were differentially expressed at day 100 compared to day 0 for H5N1 vaccination (Figure 4A), were tested for the same direction of change at day 70 compared to day 0 for H1N1-flu vaccination using a Fishers exact test. Second, for the RPs which were persistently upregulated after H5N1 vaccination (Gp 04, 06–08) enrichment was tested based on all genes ranked by the effect size at day 70 after H1N1-flu vaccination, using the fast gene set enrichment analysis R package and reporting unadjusted p values.

##### Single cell analysis at baseline

CITE-Seq data has previously been reported from the baseline timepoint for 20 recipients of the 2009–2010 pH1N1+seasonal influenza vaccine cohort, which divide equally between low and high titer responders^34^. Leading edge genes from the persistent enrichment of Gp04 and Gp06–8 at 100 days after H5N1 vaccination (Figure 4D), were assessed in the same corresponding cell types in the baseline data from the 2009-flu study, for relative expression differences with the average module z score in high compared to low responders.

### ADDITIONAL RESOURCES

This study was registered at www.clinicaltrials.gov as NCT01578317.

## Supplementary Material

1

2

## Figures and Tables

**Table:** KEY RESOURCES TABLE

REAGENT or RESOURCE	SOURCE	IDENTIFIER
Antibodies		
Flow cytometry used 30 antibodies detailed in Table S3	multiple	multiple
CITE-seq used 86 antibodies as previously described (Kotliarov et al., Nat Med, 2020)^34^	BioLegend	multiple
Biological samples		
Human peripheral blood from trial participants with characteristic described in Table S1	NCT01578317 at www.clinicaltrials.gov	N/A
Critical commercial assays		
Affymetrix Human Gene ST v2.1 Array Plate	Affymetrix	Cat# 902138
Luminex human cytokine panels detailed in Table S5	Bio-Rad	multiple
Truseq RNaseq library preparation kit	Illumina	Cat# 20020595
NovaSeq S4 200 cycle sequencing kit	Illumina	Cat# 20027466
Chromium Next GEM Single Cell 5’Library & Gel Bead Kit v1.1	10x Genomics	Cat# 1000165
Chromium Single Cell 5’ Library Construction Kit	10x Genomics	Cat# 1000020
Chromium Next GEM Single Cell ATAC Library Kit v1.1	10x Genomics	Cat# 1000163
Chromium Next GEM Single Cell ATAC Gel Bead Kit v1.1	10x Genomics	Cat# 1000159
Deposited data		
Microarray, flow cytometry, Luminex, and titer data	This paper	ImmPort (SDY1252)
All raw and analyzed data including CITE and ATAC-seq	This paper	Zenodo (https://doi.org/10.5281/zenodo.13171046)
2009-flu cohort CITE-seq	Kotliarov et al., Nat Med, 2020^34^	Figshare (https://doi.org/10.35092/yhjc.c.4753772)
Software and algorithms		
All code for this study	This paper	Zenodo (https://doi.org/10.5281/zenodo.13171046)
CellRanger	10X Genomics	https://support.10xgenomics.com/single-cell-gene-expression/software/downloads/3.1/
Seurat	Butler et al., Nat. Biotechnol., 2018^62^	https://CRAN.R-project.org/package=Seurat
Demuxlet	Kang et al., Nat. Biotechnol., 2018	https://github.com/statgen/demuxlet
dsb	Mule et al., Nat Communications, 2022	https://github.com/niaid/dsb
limma	Ritchie et al., Nucleic Acids Res, 2015^63^	https://bioconductor.org/packages/release/bioc/html/limma.html
lme4	Bates et al., J. Stat. Softw., 2015^64^	https://CRAN.R-project.org/package=lme4

## Highlights

- Unsupervised identification of immunogenicity- and adjuvant-associated response dynamics
- Adjuvant-independent early response signatures persisted to 100 days post vaccination
- Single-cell CITE-seq and ATAC-seq suggest monocytes contribute to persistent signatures
- Persistent signatures at baseline predict vaccine immunogenicity in another cohort
