# Supplementary material for: Acute and persistent responses after H5N1 vaccination in humans
Source: Cell Rep. Author manuscript; Available in PMC 2025 Mar 27. (PMC11949244; doi:10.1016/j.celrep.2024.114706)
Supplement: 1 [file NIHMS2025367-supplement-1.pdf]

Cell Reports, Volume *43*

Supplemental information

Acute and persistent responses

after H5N1 vaccination in humans

Richard Apps, Angélique Biancotto, Julián Candia, Yuri Kotliarov, Shira Perl, Foo Cheung, Rohit Farmer, Matthew P. Mulè, Nicholas Rachmaninoff, Jinguo Chen, Andrew J. Martins, Rongye Shi, Huizhi Zhou, Neha Bansal, Paula Schum, Matthew J. Olnes, Pedro Milanez-Almeida, Kyu Lee Han, Brian Sellers, Mario Cortese, Thomas Hagan, Nadine Rouphael, Bali Pulendran, Lisa King, Jody Manischewitz, Surender Khurana, Hana Golding, Robbert G. van der Most, Howard B. Dickler, Ronald N. Germain, Pamela L. Schwartzberg, and John S. Tsang

Figure S1. Quality control of gene expression-based response patterns. Related to Figure 2.

(A) To cast a wider net, one of the intermediate steps in RP construction is the addition of extra genes to the initial set with a relaxed selection criteria followed by "cleaning" (see Methods). Examples of k-mean clustering used to clean and extract core signature genes of gene expression based RPs. Each heatmap value corresponds to the correlation coefficient of a single gene (in rows) and pattern profiles for a single subject (in columns). Two examples are shown where a core signature is clearly detectable (Gp02 and Gp03), while the other two had less coherent core signatures (Gp09 and Gp10).

(B) Examples of silhouette width plots of k-means (k=2) clustering for the same gene expression based RPs as in panel A. Average silhouette width for each cluster was also used as a quality metric for each pattern (see below).

(C) Quality metrics for each gene expression RP. For each proposed RP, k-means (k=2) clustering is performed to assess whether a core, coherent set of signature genes can be identified for each RP. The following metrics, from left to right, are provided for each RP: number of genes in each cluster, average correlation coefficient for each cluster normalized to the number of genes in the cluster, average correlation distance between each pair of genes in a cluster, average silhouette distance. The genes in a subcluster with consistently higher quality metric scores (black bars) were selected as core signature genes. Two RPs with the lowest quality metrics – Gp09 and Gp10 – were excluded from elastic net modeling to predict titer outcomes (Figure 3B,G,H, Figure S5A,B).

Figure S1

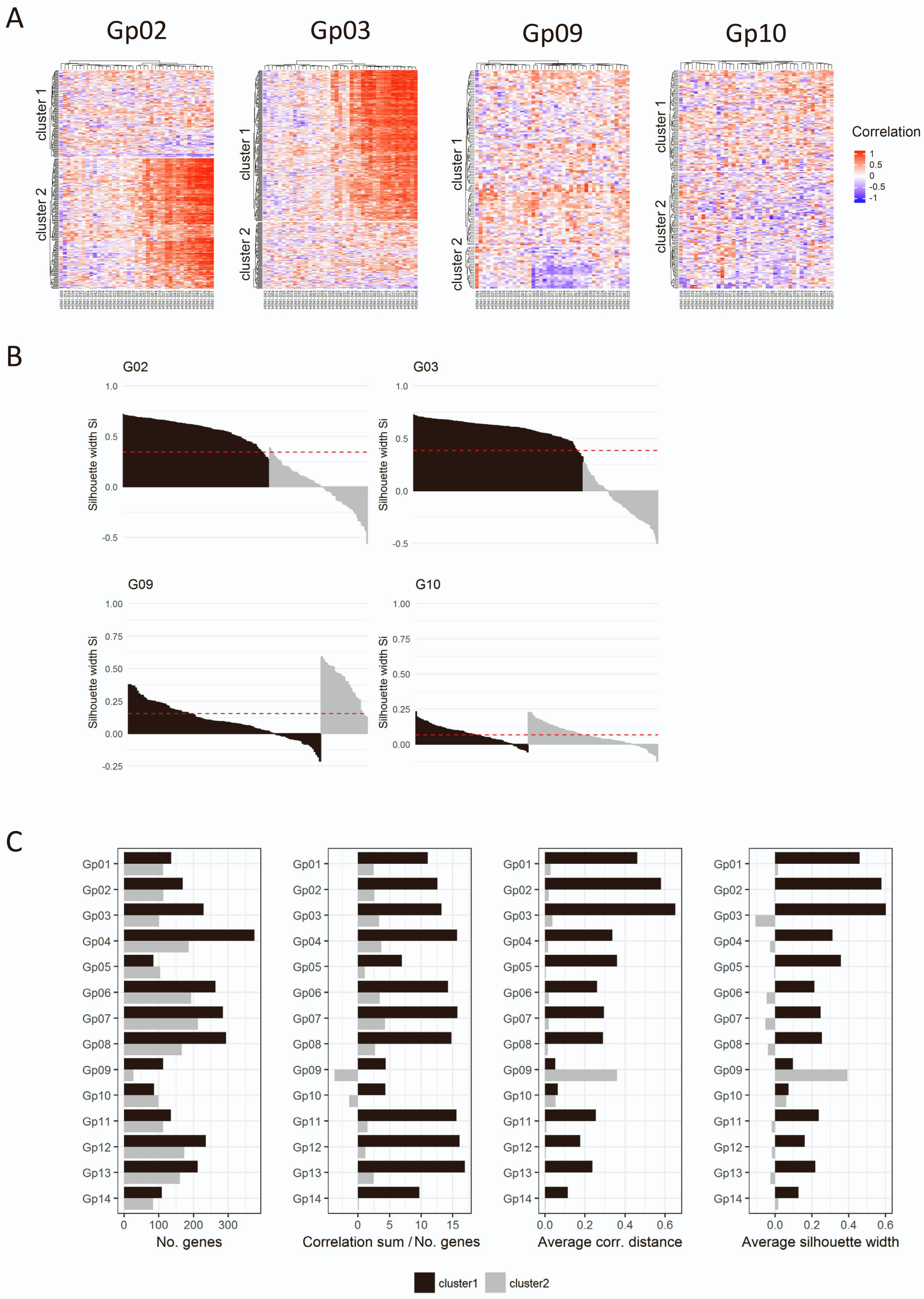
A
Gp02
Gp03
Gp09
Gp10
cluster 1
cluster 2
Correlation
1
0.5
0
-0.5
-1
B
G02
G03
G09
G10
Silhouette width Si
C
No. genes
Correlation sum / No. genes
Average corr. distance
Average silhouette width
Gp01
Gp02
Gp03
Gp04
Gp05
Gp06
Gp07
Gp08
Gp09
Gp10
Gp11
Gp12
Gp13
Gp14
cluster1
cluster2

Figure S2. Quality control of cell population frequency response patterns. Related to Figure 2.

(A) Silhouette plot (left) and average silhouette width (right) for the 8 Fp clusters.

(B) A schematic of pattern-defining cell population identification and quality control, using pattern Fp01 as an example (see also Methods). First, a cell population x subject binary matrix is generated for the response pattern in which the binary indicates whether that cell population (columns) and subject (rows) combination is assigned to Fp01. Then the Hamming distance for each pair of cell populations is calculated and the resulting matrix is clustered with k-means (k=2) to identify a coherent set of populations assigned to similar subjects. The average pattern membership normalized to the number of cell populations in a cluster is then computed for each cluster, as well as average Hamming distance and average silhouette width; these together serve as quality control metrics to assess the coherence of the response patterns. Black bars represent the selected cluster with pattern-defined cell populations.

(C) Flow cytometry population 68, CD28- CD4+ T cells, is a member of Fp01 and Fp07 although both patterns peak around 12-24 hours but only the former peaks at ~d22 (Figure 2D,E). Shown here are representative examples of 6 subjects with responses captured by Fp07 (top) or Fp01 (bottom) when the frequency of CD28- CD4+ T cells, as a percent of the parent population, is plotted longitudinally. This example shows how a parameter (in this case a cell population) can belong to more than one response pattern due to distinct dynamics in different subjects.

Figure S2

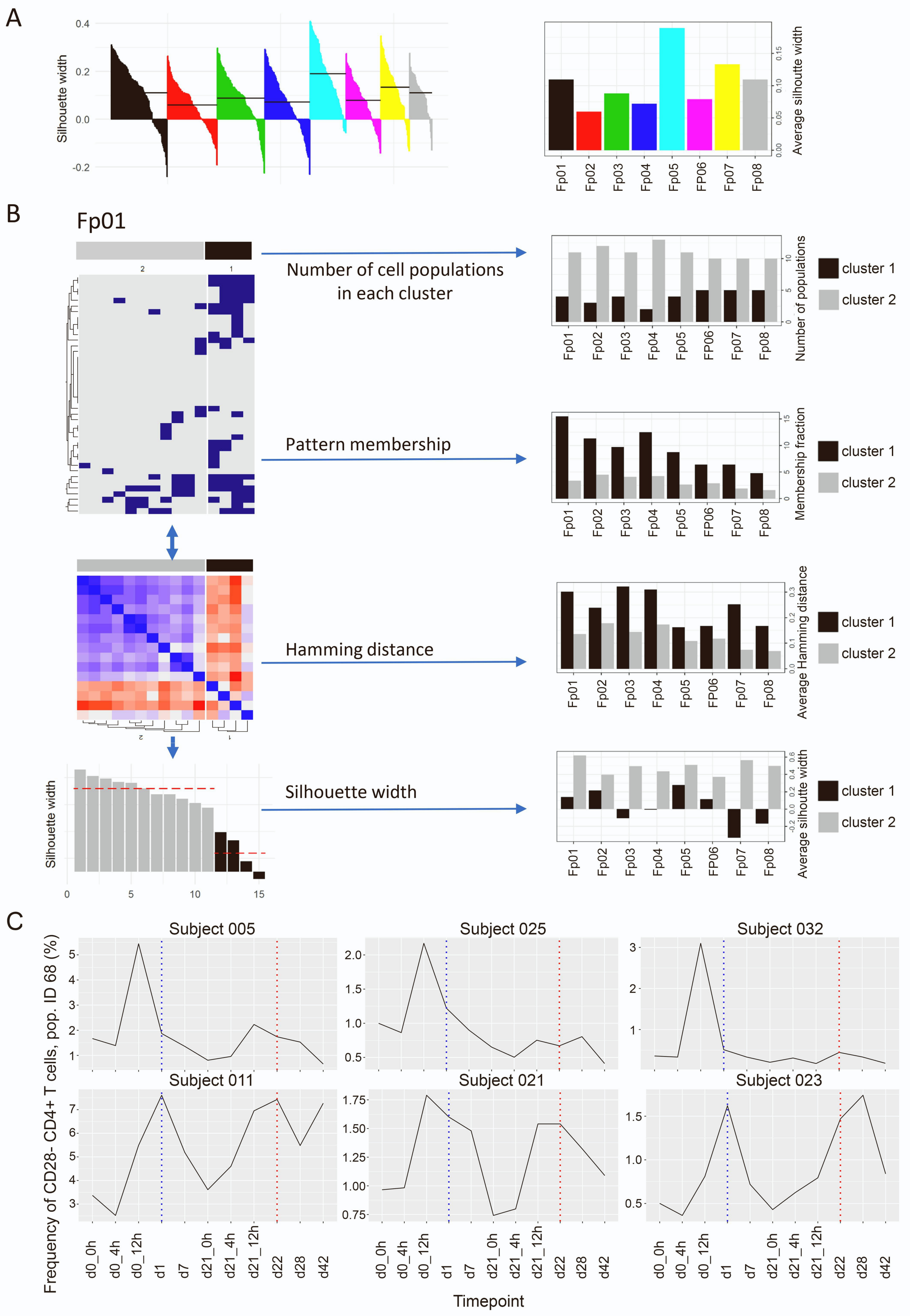
A
Silhouette width
0.4
0.2
0.0
-0.2
Average silhoutte width
0.00
0.05
0.10
0.15
Fp01
Fp02
Fp03
Fp04
Fp05
FP06
Fp07
Fp08
B
Fp01
Number of cell populations in each cluster
Number of populations
cluster 1
cluster 2
Pattern membership
Membership fraction
Hamming distance
Average Hamming distance
Silhouette width
Average silhoutte width
C
Subject 005
Subject 025
Subject 032
Subject 011
Subject 021
Subject 023
Frequency of CD28- CD4+ T cells, pop. ID 68 (%)
d0_0h
d0_4h
d0_12h
d1
d7
d21_0h
d21_4h
d21_12h
d22
d28
d42
Timepoint

Figure S3. Machine learning predictive modeling assessment using elastic net, and analyses of serum analytes used to inform adjuvant signature definition. Related to Figure 3.

(A) A cross validation based, regularized linear regression predictive modelling with elastic net is used throughout the study. This schematic outlines the approach for model training with 10-fold cross-validation of training and test sets, and how null distributions are obtained for assessing predictive parameter importance.

(B) In Figure 3C PCA separated subjects into 2 clusters based on selected RPs hypothesized to be adjuvant signatures. Between these clusters 1 (blue) and 2 (red), fold changes from day 0 to day 1 (left panels), and from day 0 to day 22 (right panels), are compared for the serum analytes CRP, MIP-1b, and SAA.

Figure S3

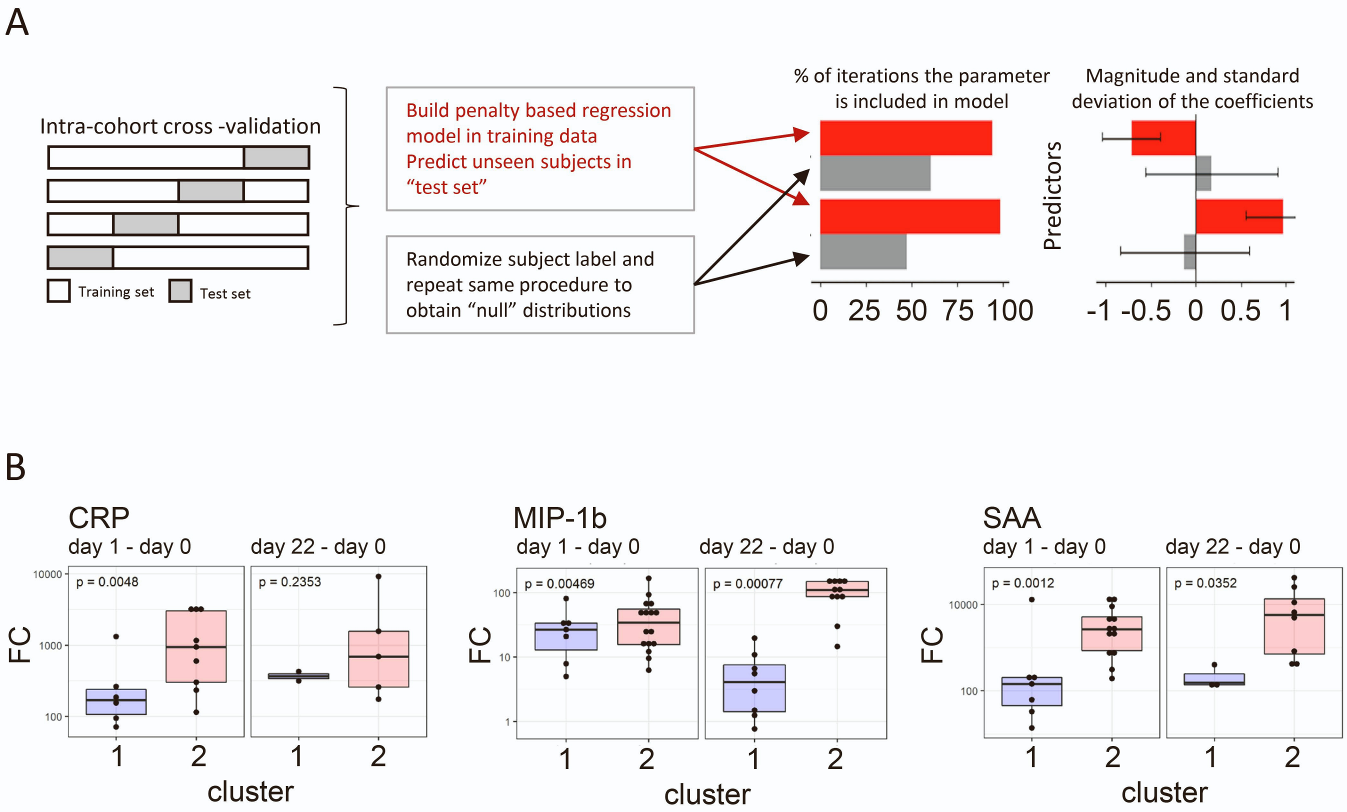
A
Intra-cohort cross -validation
Training set
Test set
Build penalty based regression model in training data
Predict unseen subjects in "test set"
Randomize subject label and repeat same procedure to obtain "null" distributions
% of iterations the parameter is included in model
0 25 50 75 100
Magnitude and standard deviation of the coefficients
Predictors
-1 -0.5 0 0.5 1
B
CRP
day 1 - day 0
day 22 - day 0
p = 0.0048
p = 0.2353
MIP-1b
p = 0.00469
p = 0.00077
SAA
p = 0.0012
p = 0.0352
FC
cluster

Figure S4. Assessment of adjuvant signature in one subject not correctly predicted at unblinding, and validation of adjuvant signatures in Emory dataset. Related to Figure 3.

(A) When unblinding was performed to assess the predicted adjuvant response signature, a single subject was classified incorrectly (Figure 3F). For this subject profiles are shown for the RP scores for gene expression and flow cytometry, as well as IP-10 levels, that comprise the adjuvant response signature.

(B) The adjuvant signature was tested for validation in an independent cohort from Emory University. Prediction of adjuvant status in the subjects from Emory was performed by combining scores from Gp1, Gp2 and Gp3 response patterns. The average score was used to rank subjects with predicted adjuvant status shown on the right in lighter colors, based on knowledge of the number of subjects known to have received the adjuvant, and similar to the threshold determined by clustering using Gp1-3 (lower red line). Actual adjuvant status is shown far-right in darker colors.

(C) Gp1, Gp2, Gp3 (left) and IP-10 (right) profiles are shown for two representative Emory subjects that were incorrectly predicted.

Figure S4

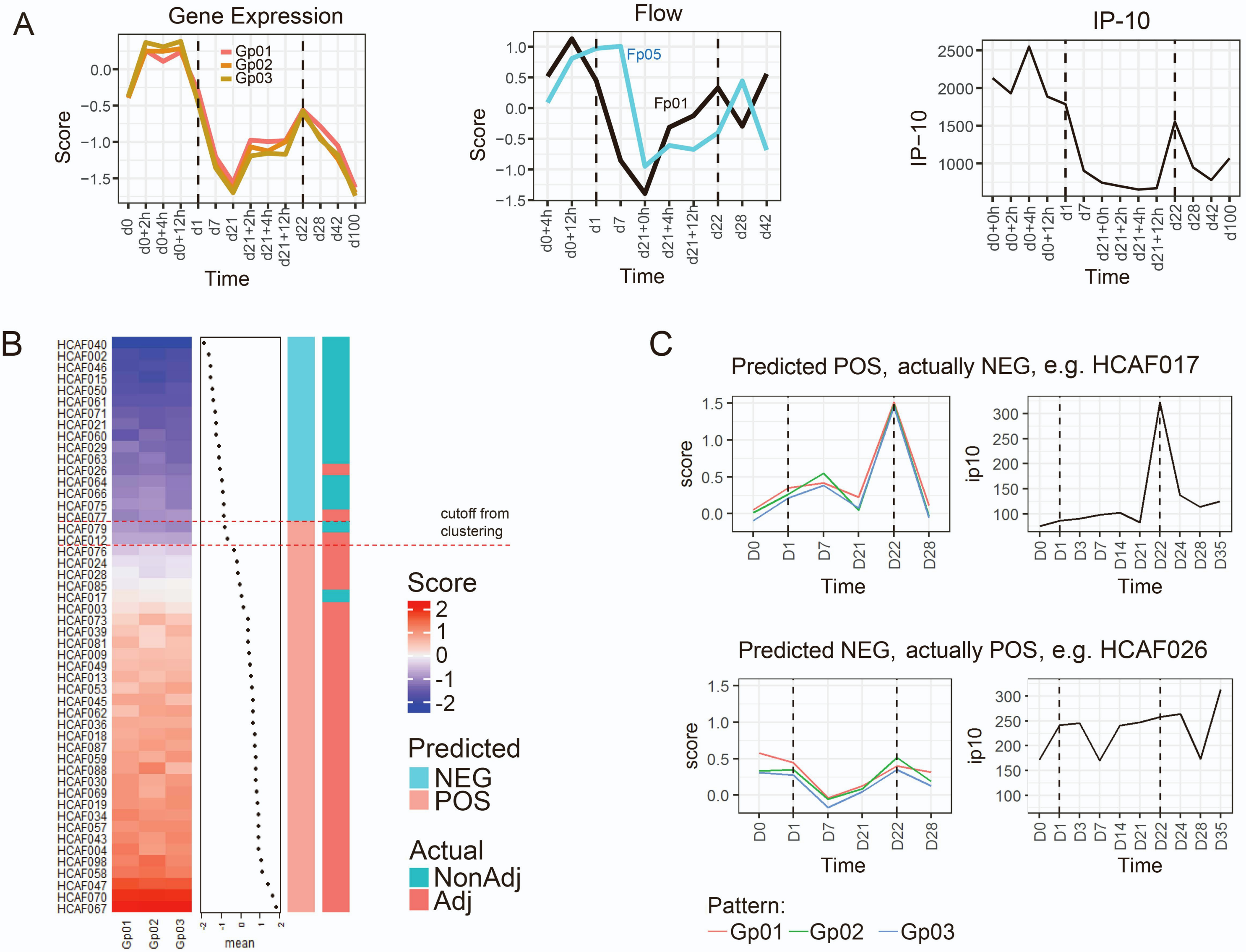
A
Gene Expression
Gp01
Gp02
Gp03
Score
Time
Flow
Fp05
Fp01
Score
Time
IP-10
IP−10
Time
B
cutoff from clustering
Score
Predicted
NEG
POS
Actual
NonAdj
Adj
mean
C
Predicted POS, actually NEG, e.g. HCAF017
score
ip10
Time
Predicted NEG, actually POS, e.g. HCAF026
score
ip10
Time
Pattern:
Gp01
Gp02
Gp03

Figure S5. Regularized linear modeling using adjuvant status predicted by adjuvant associated signature. Related to Figure 3.

(A,B) In Figure 3G and 3H, RPs were used to model titer responses in adjuvant and non-adjuvanted subjects. Here equivalent EN modeling is shown using predicted adjuvant status for POS and NEG subjects, respectively, based on the adjuvant associated signature. Subplots are as described for Figure 3B.

Figure S5

A Modeling titer response for predicted Adj donors using response profiles

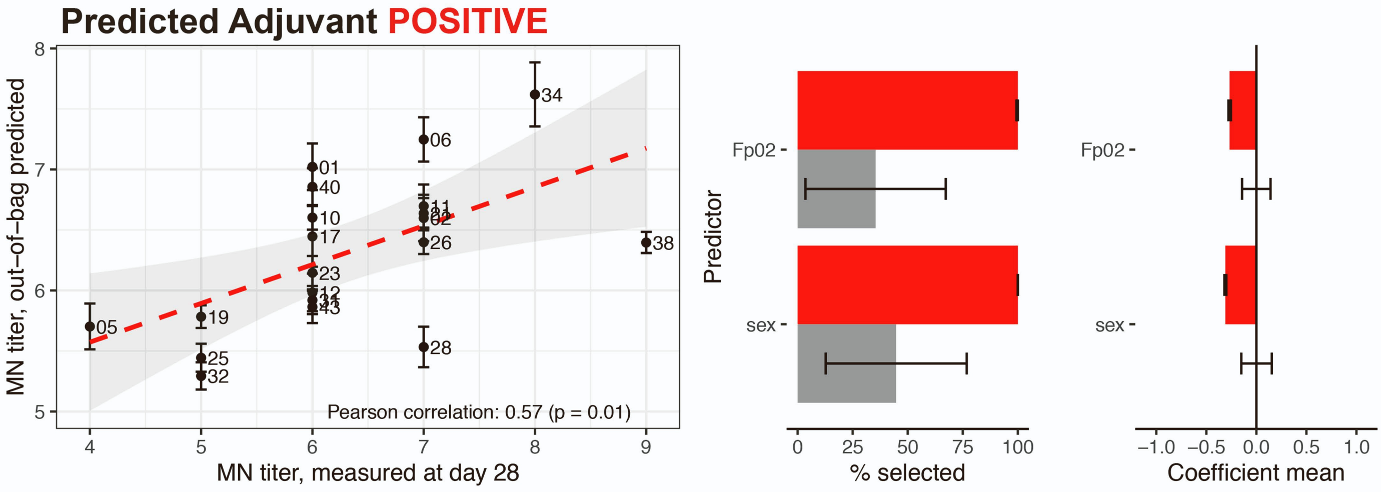

B Modeling titer response for predicted NonAdj donors using response profiles

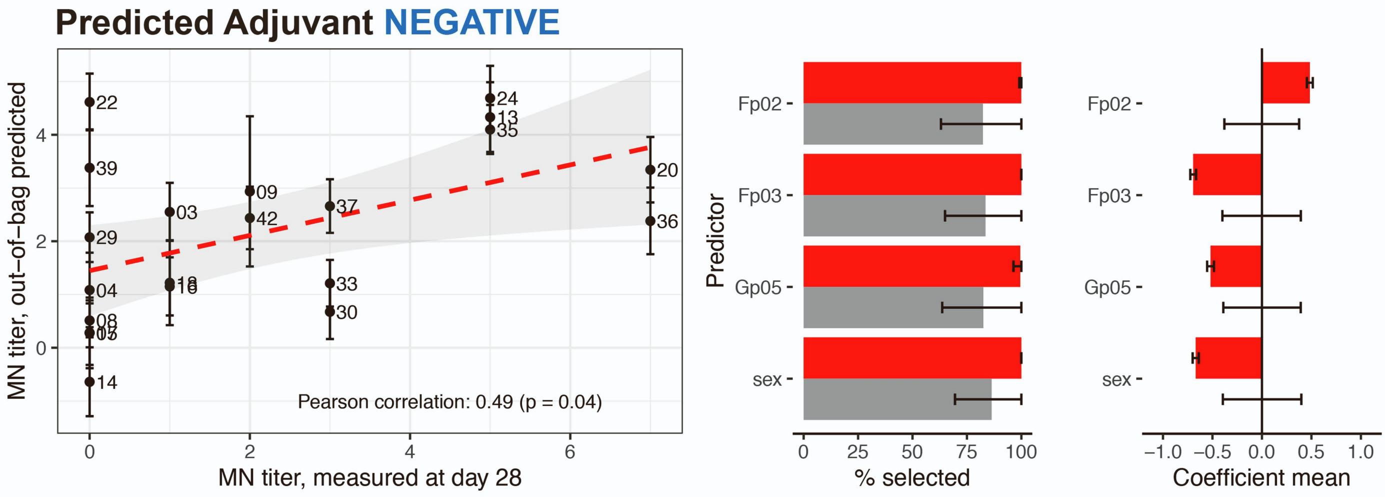

Figure S6. Analysis of persistent patterns that remain different from baseline at day 100 after H5N1 vaccination using bulk gene expression data. Related to Figure 4.

(A) As in Figure 4B, hypergeometric test to assess BTM enrichment of differentially expressed RP genes from day 100 after H5N1 vaccination compared to baseline is shown here for individual RPs in all subjects, or for combined RPs in either adjuvanted or unadjuvanted subjects.

(B) For the genes in each persistent RP, baseline vs day 100 log fold change in gene expression is plotted for subjects receiving adjuvanted (x-axis) or non-adjuvanted vaccine (y-axis), with Pearson correlation coefficient and p-value shown.

Figure S6

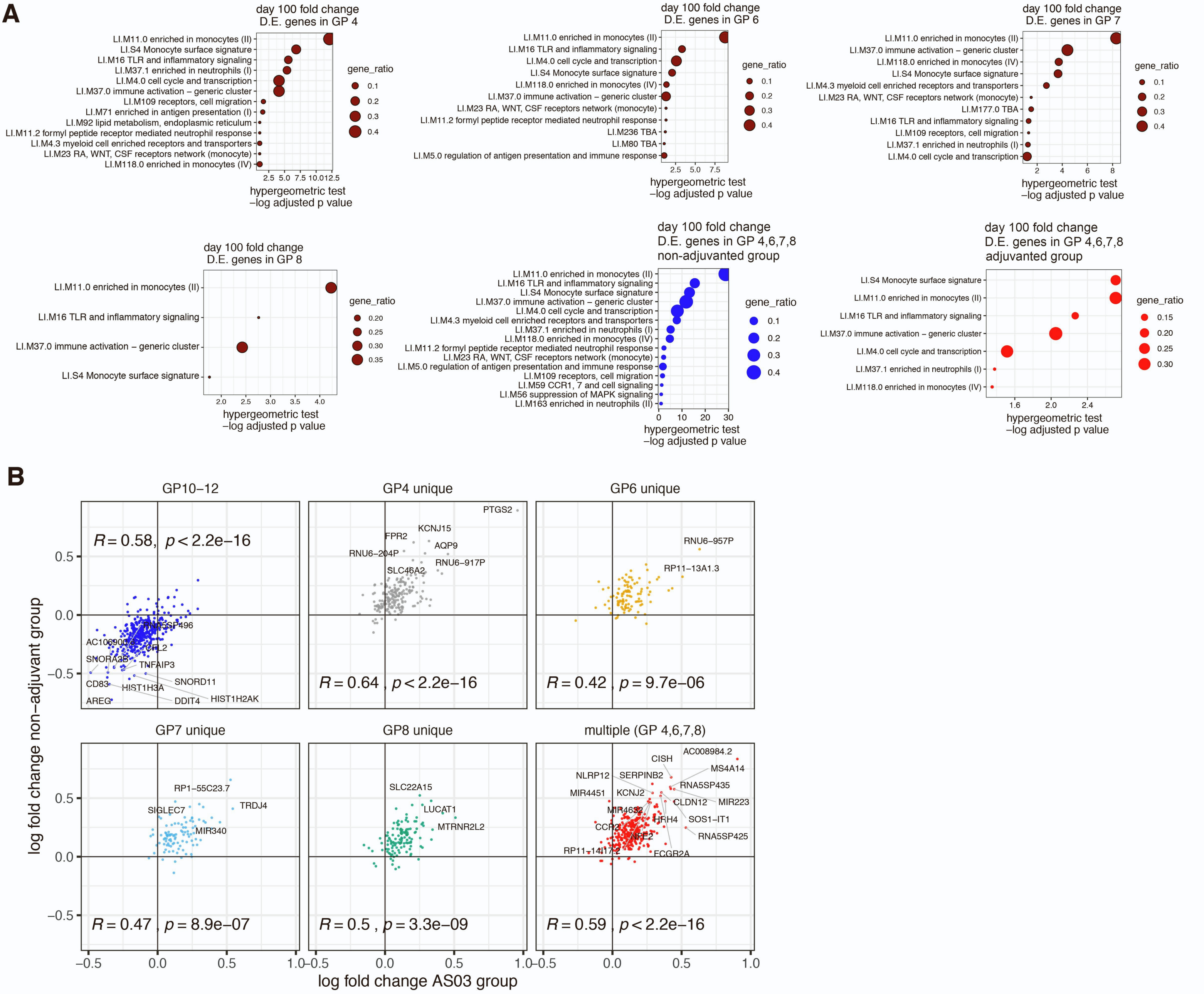
A
day 100 fold change
D.E. genes in GP 4
LI.M11.0 enriched in monocytes (II)
LI.S4 Monocyte surface signature
LI.M16 TLR and inflammatory signaling
LI.M37.1 enriched in neutrophils (I)
LI.M4.0 cell cycle and transcription
LI.M37.0 immune activation – generic cluster
LI.M109 receptors, cell migration
LI.M71 enriched in antigen presentation (I)
LI.M92 lipid metabolism, endoplasmic reticulum
LI.M11.2 formyl peptide receptor mediated neutrophil response
LI.M4.3 myeloid cell enriched receptors and transporters
LI.M23 RA, WNT, CSF receptors network (monocyte)
LI.M118.0 enriched in monocytes (IV)
gene_ratio
hypergeometric test
–log adjusted p value
day 100 fold change
D.E. genes in GP 6
LI.M236 TBA
LI.M80 TBA
LI.M5.0 regulation of antigen presentation and immune response
day 100 fold change
D.E. genes in GP 7
LI.M177.0 TBA
day 100 fold change
D.E. genes in GP 8
day 100 fold change
D.E. genes in GP 4,6,7,8
non-adjuvanted group
LI.M109 receptors, cell migration
LI.M59 CCR1, 7 and cell signaling
LI.M56 suppression of MAPK signaling
LI.M163 enriched in neutrophils (II)
day 100 fold change
D.E. genes in GP 4,6,7,8
adjuvanted group
B
GP10–12
R = 0.58, p < 2.2e–16
GP4 unique
R = 0.64 , p < 2.2e–16
GP6 unique
R = 0.42 , p = 9.7e–06
GP7 unique
R = 0.47 , p = 8.9e–07
GP8 unique
R = 0.5 , p = 3.3e–09
multiple (GP 4,6,7,8)
R = 0.59 , p < 2.2e–16
log fold change non–adjuvant group
log fold change AS03 group

Figure S7. Supporting data for the single cell analysis of persistent signatures in the H5N1 cohort. Related to Figure 4.

(A) CITE-seq for n=6 adjuvanted subjects is described in Figure 4C. Here a heatmap of the average surface protein expression profile is shown for each of the major cell cluster/types annotated in Figure 4C.

(B) CITE-seq for a further n=10 adjuvanted subjects shows similar persistent effects to those observed for n=6 adjuvanted subjects in Figure 4D. Initially n=6 adjuvanted subjects were analysed by CITE-seq at days 0 and 100, showing persistent effects at d100 were enriched for Gp4,6-8 particularly in CD14+ monocytes and cd8+ naïve T cells (Figure 4D). Subsequently a further 13 subjects were analyzed by CITE-seq, of these 10 were adjuvanted and here these show similar persistent effects of Gp4,6-8 in CD14+ monocytes and CD8+ naïve T cells. For this CITE-seq dataset persistent effects were assessed by comparing d100 to d21 (day of dose 2), as at d21 these RPs had previously been determined to decline to d0 levels (Figure 2B).

(C) scATAC-seq for n=13 subjects identified transcription factors with similar differentially accessibility both immediately and at persistently after dose 2, as described in Figure 4F but here showing results for CD8+ naïve T cells. Differential accessibility is shown for all Motifs enriched near the d100 LE genes which had been observed for CD8+ naïve T cells in Figure 4D and were identified using centrimo. The differential accessibility of these motifs was then determined by computing chromVAR accessibility scores and linear mixed effect models. Shown is the scaled effect size and 95% confidence interval (1.96*std.err.).

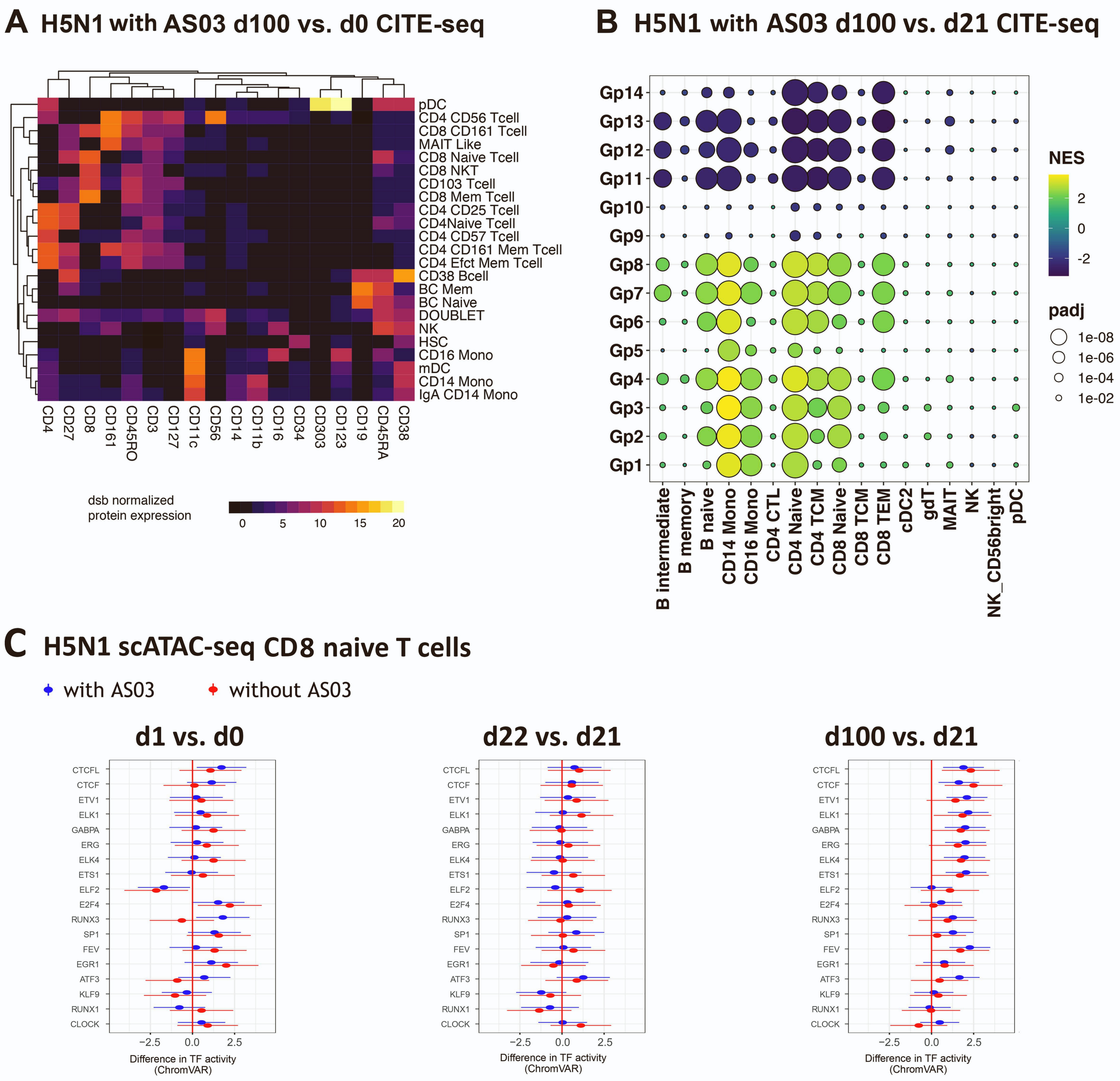
A H5N1 with AS03 d100 vs. d0 CITE-seq
pDC
CD4 CD56 Tcell
CD8 CD161 Tcell
MAIT Like
CD8 Naive Tcell
CD8 NKT
CD103 Tcell
CD8 Mem Tcell
CD4 CD25 Tcell
CD4Naive Tcell
CD4 CD57 Tcell
CD4 CD161 Mem Tcell
CD4 Efct Mem Tcell
CD38 Bcell
BC Mem
BC Naive
DOUBLET
NK
HSC
CD16 Mono
mDC
CD14 Mono
IgA CD14 Mono
CD4 CD27 CD8 CD161 CD45RO CD3 CD127 CD11c CD56 CD14 CD11b CD16 CD34 CD303 CD123 CD19 CD45RA CD38
dsb normalized protein expression
0 5 10 15 20
B H5N1 with AS03 d100 vs. d21 CITE-seq
Gp14 Gp13 Gp12 Gp11 Gp10 Gp9 Gp8 Gp7 Gp6 Gp5 Gp4 Gp3 Gp2 Gp1
B intermediate B memory B naive CD14 Mono CD16 Mono CD4 CTL CD4 Naive CD4 TCM CD8 Naive CD8 TCM CD8 TEM cDC2 gdT MAIT NK NK_CD56bright pDC
NES
2
0
-2
padj
1e-08
1e-06
1e-04
1e-02
C H5N1 scATAC-seq CD8 naive T cells
with AS03
without AS03
d1 vs. d0
d22 vs. d21
d100 vs. d21
CTCFL CTCF ETV1 ELK1 GABPA ERG ELK4 ETS1 ELF2 E2F4 RUNX3 SP1 FEV EGR1 ATF3 KLF9 RUNX1 CLOCK
−2.5 0.0 2.5
Difference in TF activity (ChromVAR)

Figure S8. Supporting data for investigation of the persistent signatures observed in the H5N1 cohort in an independent cohort of 2009-2010 pH1N1+seasonal influenza vaccination. Related to Figure 4.

(A) Using bulk transcriptomic data all genes from persistently altered response patterns differential expressed at day 100 compared to day 0 for H5N1 vaccination (see Figure 4A) were tested for the direction of change in these genes at day 70 compared to day 0 for the 2009-2010 pH1N1+seasonal influenza vaccine cohort using a Fisher's exact test.

(B) Using bulk transcriptomic data persistently altered genes were detected in the independent cohort of 2009-2010 pH1N1+seasonal influenza vaccination by comparing PBMC gene expression between day 70 post vaccination vs. baseline (day 0). These genes were ranked by their changes in expression at day 70 compared to day 0, followed by GSEA analysis of the persistent genes from the indicated RPs identified from the H5N1 cohort. Normalised enrichment scores are shown.

(C) Using CITE-seq data analysed as in Figure 4F, relative expression compared at baseline between high and low antibody responders in the 2009-flu cohort is shown in corresponding cell types for leading edge genes from the day 100 differentially expressed gene patterns enriched in CD8+ naïve-like T cells, monocytes, and dendritic cells. Here showing RP-cell type combinations not shown in Figure 4F, as well as for all RPs combined for each cell type.

Figure S8

A

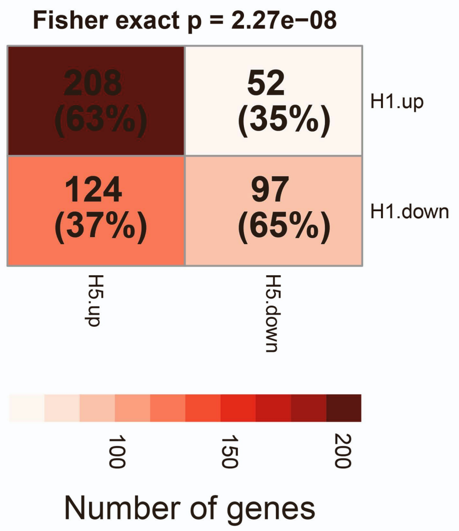

B

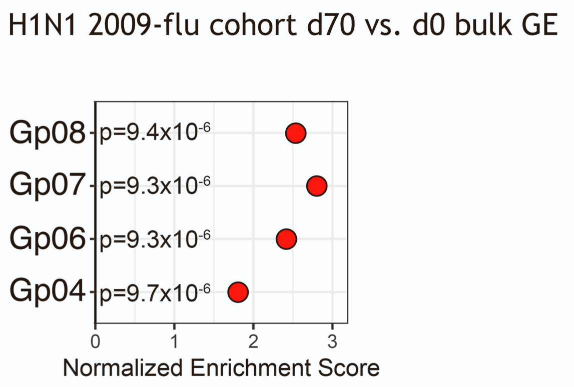

C

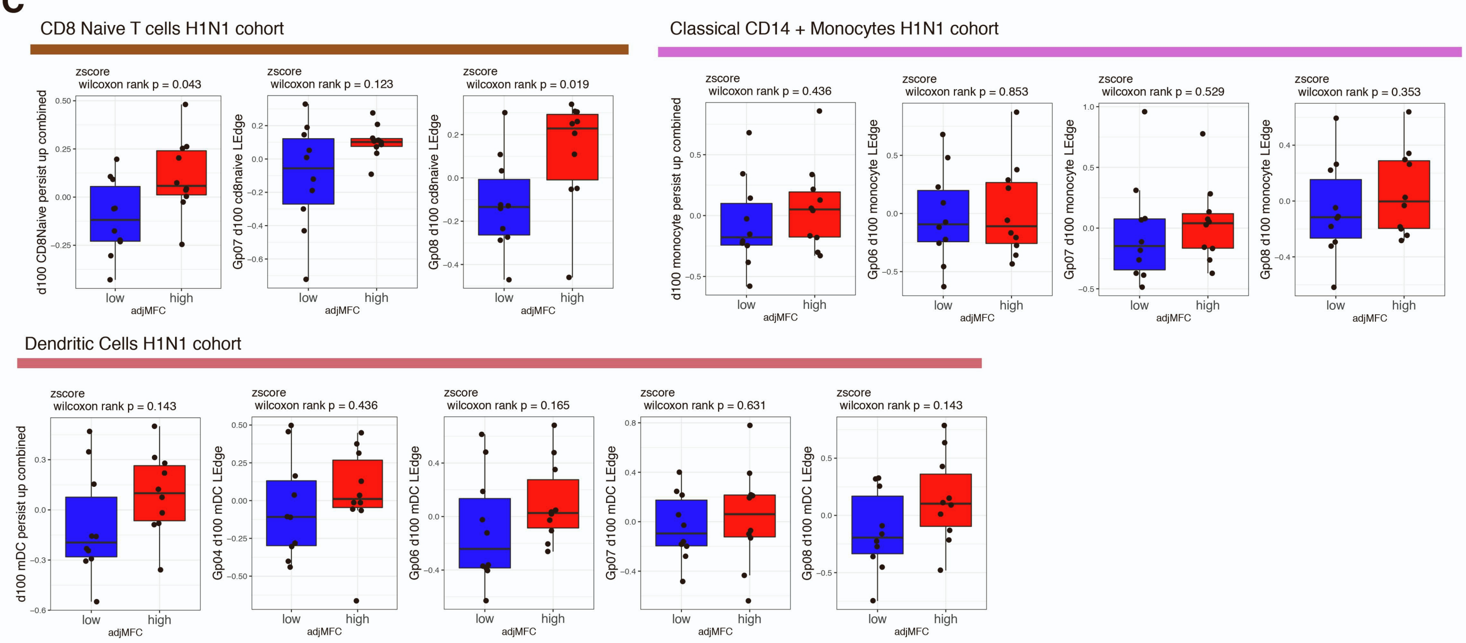

Figure S9. Analyses of titer responses in this H5N1 vaccine cohort. Related to STAR Methods.

(A) The percentage response rate is shown for adjuvanted and non-adjuvanted subjects at days 21, 28 and 42. Responders are those with at least a 4-fold increase from baseline in microneutralization (MN) titers to the A/Indonesia strain.

(B) HAI titer profiles for all subjects at all time points.

(C) Distribution of adjuvanted and non-adjuvanted subjects by time point at which the MN titer reaches its maximum value for a particular subject.

(D) Scatter plot of percentage decline of MN titer value after its peak time against the peak value for adjuvanted and non-adjuvanted subjects. The plot shows lower peak values and faster decline for the non-adjuvanted subjects in comparison to adjuvanted subjects.

Figure S9

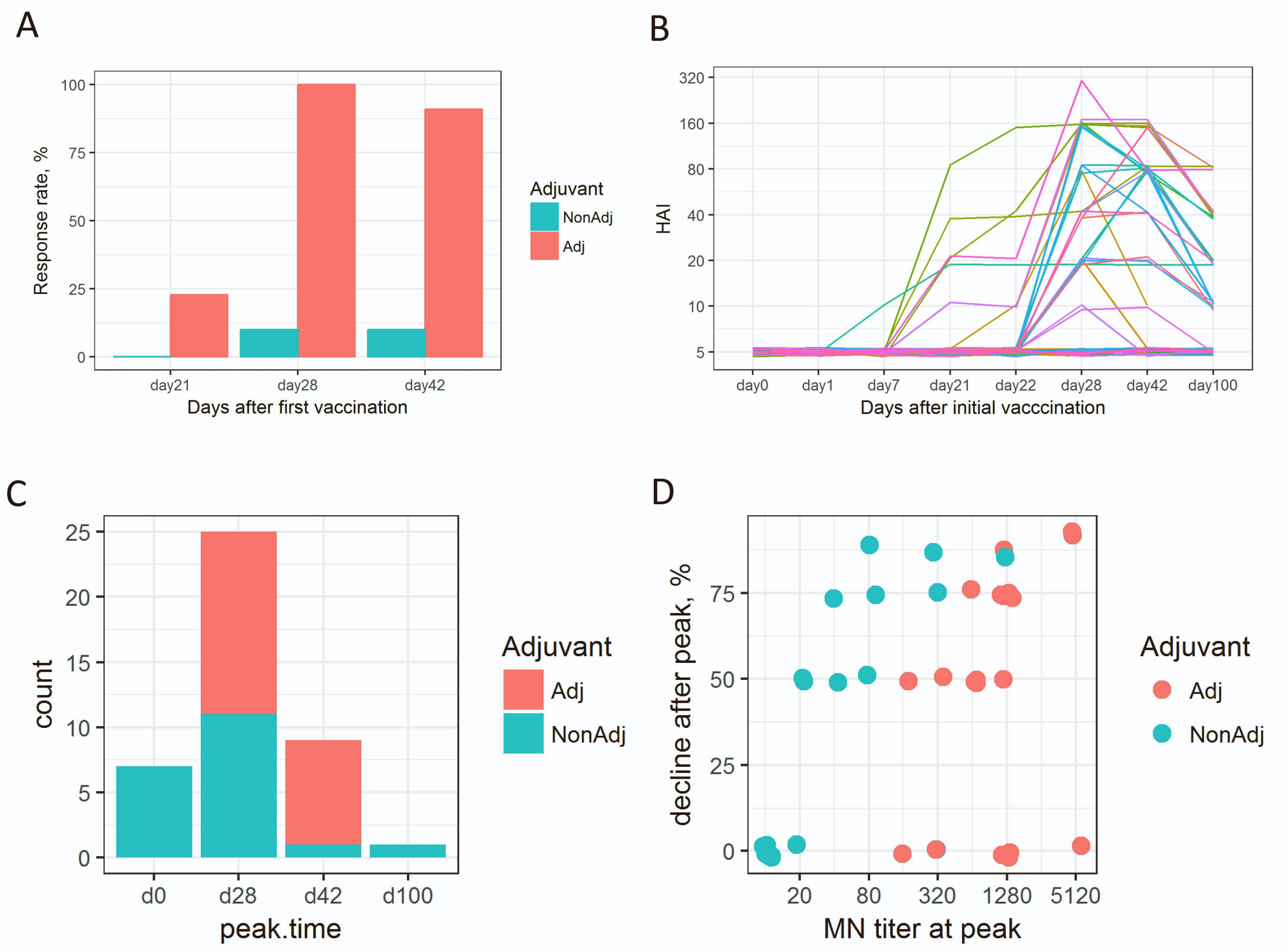
A
Response rate, %
100
75
50
25
0
day21
day28
day42
Days after first vaccination
Adjuvant
NonAdj
Adj
B
HAI
320
160
80
40
20
10
5
day0
day1
day7
day21
day22
day28
day42
day100
Days after initial vacccination
C
count
25
20
15
10
5
0
d0
d28
d42
d100
peak.time
Adjuvant
Adj
NonAdj
D
decline after peak, %
75
50
25
0
20
80
320
1280
5120
MN titer at peak
Adjuvant
Adj
NonAdj

Table S1. Demographic information for the subjects studied and for each time point sampled the immunological assays performed. Related to Figure 1. L = Serum protein by Luminex; A = Gene expression microarray from PBMC; F10 = 10 color flow cytometry.

	Demographics			Vaccination		Time point													
Subject ID	Gender	Age	Self-reported ethinicity	Timeframe	ASO3	Day0-0h	Day0-2h	Day0-4h	Day0-12h	Day1	Day7	Day21-0h	Day21-2h	Day21-4h	Day21-12h	Day22	Day28	Day42	Day100
H5N1-001	Male	44	African American	June-13	Y	L A F10	L A	L A F10	L A F10	L A F10	L A F10	L A F10	L A	L A F10	L A F10	L A F10	L A F10	L A F10	L A
H5N1-002	Female	35	White	June-13	Y	L A F10	L A	L A F10	L A F10	L A F10	L A F10	A F10	A	A F10	A F10	A F10	L A F10	L A F10	L A
H5N1-003	Female	24	Multiracial	June-13	N	L A F10	L A	L A F10	L A F10	L A F10	L A F10	A F10	L A	L A F10	L A F10	L A F10	L A F10	L A F10	L A
H5N1-004	Female	34	White	June-13	N	L A F10	L A	L A F10	L A F10	L A F10	L A F10	L A F10	L A	L A F10	L A F10	L A F10	L A F10	L A F10	L A
H5N1-005	Male	25	African American	June-13	Y	L A F10	L A	L A F10	L A F10	L A F10	L A F10	L A F10	L A	L A F10	L A F10	L A F10	L A F10	L A F10	L A
H5N1-006	Female	28	Multiracial	June-13	Y	L A F10	L A	L A F10	L A F10	L A F10	L A F10	L A F10	L A	L A F10	L A F10	L A F10	L A F10	L A F10	L A
H5N1-007	Female	31	Asian	June-13	N	L A F10	L A	L A F10	L A F10	L A F10	L A F10	L A F10	L A	L A F10	L A F10	L A F10	L A F10	L A F10	L A
H5N1-008	Female	38	White	June-13	N	L A F10	L A	L A F10	L A F10	L A F10	L A F10	L A F10	L A	L A F10	L A F10	L A F10	L A F10	L A F10	L A
H5N1-009	Male	30	White	July-13	N	L A F10	L A	L A F10	L A F10	L A F10	L A F10	L A F10	L A	L A F10	L A F10	L A F10	L A F10	L A F10	L A
H5N1-010	Female	43	White	July-13	Y	L A F10	L A	L A F10	L A F10	L A F10	L A F10	L A F10	L A	L A F10	L A F10	L A F10	L A F10	L A F10	L A
H5N1-011	Female	43	White	July-13	Y	L A F10	L A	L A F10	L A F10	L A F10	L A F10	L A F10	L A	L A F10	L A F10	L A F10	L A F10	L A F10	L A
H5N1-012	Female	25	African American	July-13	Y	L A F10	L A	L A F10	L A F10	L A F10	L A F10	L A F10	L A	L A F10	L A F10	L A F10	L A F10	L A F10	L A
H5N1-013	Female	35	White	July-13	N	L A F10	L A	L A F10	L A F10	L A F10	L A F10	A F10	A	A F10	A F10	A F10	L A F10	L A F10	L A
H5N1-014	Male	38	White	July-13	N	L A F10	L A	L A F10	L A F10	L A F10	L A F10	L A F10	L A	L A F10	L A F10	L A F10	L A F10	L A F10	L A
H5N1-015	Male	38	White	July-13	N	L A F10	L A	L A F10	L A F10	L A F10	L A F10	L A F10	L A	L A F10	L A F10	L A F10	L A F10	L A F10	L A
H5N1-016	Female	24	Asian	July-13	N	L A F10	L A	L A F10	L A F10	L A F10	L A F10	L A F10	L A	L A F10	L A F10	L A F10	L A F10	L A F10	L A
H5N1-017	Male	27	White	July-13	Y	L A F10	L A	L A F10	L A F10	L A F10	L A F10	L A F10	L A	L A F10	L A F10	L A F10	L A F10	L A F10	L A
H5N1-018	Female	34	White	July-13	N	L A F10	L A	L A F10	L A F10	L A F10	L A F10	L A F10	L A	L A F10	L A F10	L A F10	L A F10	L A F10	L A
H5N1-019	Male	39	African American	July-13	Y	L A F10	L A	L A F10	L A F10	L A F10	L A F10	L A F10	L A	L A F10	L A F10	L A F10	L A F10	L A F10	L A
H5N1-020	Female	27	White	July-13	Y	L A F10	L A	L A F10	L A F10	L A F10	L A F10	L A F10	L A	L A F10	L A F10	L A F10	L A F10	L A F10	L A
H5N1-021	Female	32	White	July-13	Y	L A F10	L A	L A F10	L A F10	L A F10	L A F10	L A F10	L A	L A F10	L A F10	L A F10	L A F10	L A F10	L A
H5N1-022	Male	34	White	July-13	N	L A F10	L A	L A F10	L A F10	L A F10	L A F10	L A F10	L A	L A F10	L A F10	L A F10	L A F10	L A F10	L A
H5N1-023	Female	31	White	August-13	Y	L A F10	L A	L A F10	L A F10	L A F10	L A F10	L A F10	L A	L A F10	L A F10	L A F10	L A F10	L A F10	L A
H5N1-024	Female	40	African American	August-13	N	L A F10	L A	L A F10	L A F10	L A F10	L A F10	L A F10	L A	L A F10	L A F10	L A F10	L A F10	L A F10	L A
H5N1-025	Male	32	White	August-13	Y	L A F10	L A	L A F10	L A F10	L A F10	L A F10	L A F10	L A	L A F10	L A F10	L A F10	L A F10	L A F10	L A
H5N1-026	Female	32	White	August-13	Y	L A F10	L A	L A F10	L A F10	L A F10	L A F10	L A F10	L A	L A F10	L A F10	L A F10	L A F10	L A F10	L A
H5N1-028	Male	31	Native Hawaiian	September-13	Y	L A F10	L A	L A F10	L A F10	L A F10	L A F10	L A F10	L A	L A F10	L A F10	L A F10	L A F10	L A F10	L A
H5N1-029	Male	26	Asian	August-13	N	L A F10	L A	L A F10	L A F10	L A F10	L A F10	L A F10	L A	L A F10	L A F10	L A F10	L A F10	L A F10	L A
H5N1-030	Male	23	White	September-13	N	L A F10	L A	L A F10	L A F10	L A F10	L A F10	L A F10	L A	L A F10	L A F10	L A F10	L A F10	L A F10	L A
H5N1-031	Male	40	White	September-13	Y	L A F10	L A	L A F10	L A F10	L A F10	L A F10	L A F10	L A	L A F10	L A F10	L A F10	L A F10	L A F10	L A
H5N1-032	Male	41	White	September-13	Y	L A F10	L A	L A F10	L A F10	L A F10	L A F10	A F10	A	A F10	A F10	A F10	L A F10	L A F10	L A
H5N1-033	Female	27	African American	September-13	N	L A F10	L A	L A F10	L A F10	L A F10	L A F10	L A F10	L A	L A F10	L A F10	L A F10	L A F10	L A F10	L A
H5N1-034	Female	38	White	September-13	Y	L A F10	L A	L A F10	L A F10	L A F10	L A F10	L A F10	L A	L A F10	L A F10	L A F10	L A F10	L A F10	L A
H5N1-035	Female	25	African American	September-13	N	L A F10	L A	L A F10	L A F10	L A F10	L A F10	L A F10	L A	L A F10	L A F10	L A F10	L A F10	L A F10	L A
H5N1-036	Female	33	White	September-13	N	L A F10	L A	L A F10	L A F10	L A F10	L A F10	L A F10	L A	L A F10	L A F10	L A F10	L A F10	L A F10	L A
H5N1-037	Male	32	African American	September-13	N	L A F10	L A	L A F10	L A F10	L A F10	L A F10	L A F10	L A	L A F10	L A F10	L A F10	L A F10	L A F10	L A
H5N1-038	Female	25	African American	September-13	Y	L A F10	L A	L A F10	L A F10	L A F10	L A F10	A F10	A	A F10	A F10	A F10	A F10	L A F10	L A
H5N1-039	Female	29	White	October-13	N	L A F10	L A	L A F10	L A F10	L A F10	L A F10	A F10	A	L A F10	A F10	A F10	L A F10	L A F10	L A
H5N1-040	Female	28	White	October-13	Y	L A F10	L A	L A F10	L A F10	L A F10	L A F10	L A F10	L A	L A F10	L A F10	L A F10	L A F10	L A F10	L A
H5N1-042	Male	22	White	November-13	N	L A F10	L A	L A F10	L A F10	L A F10	L A F10	L A F10	L A	L A F10	L A F10	L A F10	L A F10	L A F10	L A
H5N1-043	Male	30	Multiracial	November-13	Y	L A F10	L A	L A F10	L A F10	L A F10	L A F10	A F10	A	A F10	A F10	A F10	L A F10	L A F10	L A
H5N1-044	Female	43	African American	January-14	Y	L A F10	L A	L A F10	L A F10	L A F10	L A F10	L A F10	L A	L A F10	L A F10	L A F10	L A F10	L A F10	L A

Table S3. Five parallel 10 color staining panels used for flow cytometry. Related to STAR Methods. Antigens are shown in bold, with antibody clones in italics. Markers in black were prepared in a single batch of lyophilized plates, with those shown in green added as liquid reagents on each day of experiment.

			HIPc-10c				
			T lineage			B lineage	Myeloid lineage
**Excitation wavelength**	**Fluorochrome**	**PMT name**	**T cell**	**Treg**	**T helper**	**B cell**	**DC/Mono/NK**
355 nm	BUV395	V450	**CD45**	**CD45**	**CD45**	**CD45**	**CD45**
			HI30	*HI30*	*HI30*	*HI30*	*HI30*
	UV450	V545	**Viability**	**Viability**	**Viability**	**Viability**	**Viability**
407 nm	V450	V605	**CD3**	**CD3**	**CD3**	**CD3**	**CD14**
			UCHT1	*UCHT1*	*UCHT1*	*UCHT1*	*M F P9*
	V500	V655	**HLA-DR**	**HLA-DR**	**HLA-DR**	**IgD**	**HLA-DR**
			G46-6	*G46-6*	*G46-6*	*IA6-2*	*G46-6*
488 nm	FITC/BB515	B515	**CD28**	**CD39**	**CXCR5**	**CD10**	**CD163**
			CD28.2	*Tu66*	*RF8B2*	*HI10 a*	*GHI/61*
	PerCP-Cy5.5	B710	**CD4**	**CD4**	**CD4**	**CD19**	**CD123**
			SK3	*SK3*	*SK3*	*SJ25C1*	*7G3*
532 nm	PE	G560	**CD197**	**CD25**	**CXCR3**	**CD24**	**CD56**
			150503	*2A3*	*1C6*	*ML5*	*B159*
	PE-Cy7	G780	**CD45-RA**	**CCR4**	**CCR6**	**CD27**	**CD11c**
			L48	*1G1*	*11A9*	*M-T271*	*B-Ly6*
633 nm	APC/Alexa Fluor 647	R660	**CD38**	**CD127**	**CD38**	**CD38**	**CD16**
			HIT2	*hIL-7R-M21*	*HIT2*	*HIT2*	*B73.1*
	APC-H7	R780	**CD8**	**CD45-RO**	**CD8**	**CD20**	**CD3+CD19+CD20+**
			SK1	*UCHL1*	*SK1*	*2H7*	*SK7+SJ25C1+2H7*

Table S4. Populations of PBMC quantified by flow cytometry analysis. Related to STAR Methods. Gating hierarchies used to define 84 populations and the parent populations used to quantify the frequency of these populations.

Tube	Pop Code	Gate 1	Gate 2	Gate 3	Gate 4	Gate 5	Parent population for expressing frequency of defined population	Subset name	Subset name 2
T-cell	1.1	CD3					viable mononuclear cells	T cells	CD3+ viable mononuclear cells
	2.1	CD3	CD4+/CD8+				CD3	DP T cells	CD4+CD8+ T cells
	3.1	CD3	CD4-/CD8-				CD3	DN T cells	CD4-CD8- T cells
	4.1	CD3	CD4+/CD8-				CD3	T helper	CD4+CD8- T cells
	5.1	CD3	CD4+/CD8-	HLA-DR+/CD38+			CD3/CD4+/CD8-	CD38+HLA-DR+ activated T helper	
	6.1	CD3	CD4+/CD8-	CD38+			CD3/CD4+/CD8-	CD38+ activated T helper	
	7.1	CD3	CD4+/CD8-	HLA-DR+			CD3/CD4+/CD8-	HLA-DR+ activated T helper	
	8	CD3	CD4+/CD8-	CD45RA+			CD3/CD4+/CD8-	CD45RA+ T helper	
	9	CD3	CD4+/CD8-	CD45RA-			CD3/CD4+/CD8-	CD45RA- T helper	
	10	CD3	CD4+/CD8-	CD28+	CCR7+/CD45RA+		CD3/CD4+/CD8-/CD28+	naïve T helper	CD45RA+CCR7+ T helper
	11	CD3	CD4+/CD8-	CD28+	CCR7+/CD45RA-		CD3/CD4+/CD8-/CD28+	central memory T helper	CD45RA-CCR7+ T helper
	12	CD3	CD4+/CD8-	CD28+	CCR7-/CD45RA-		CD3/CD4+/CD8-/CD28+	effector memory T helper	CD45RA-CCR7- T helper
	13	CD3	CD4+/CD8-	CD28+	CCR7-/CD45RA+		CD3/CD4+/CD8-/CD28+	effector T helper	CD45RA+CCR7-T helper
	14.1	CD3	CD8+/CD4-				CD3	T c/s	CD4-CD8+ T cells
	15.1	CD3	CD8+/CD4-	HLA-DR+/CD38+			CD3/CD8+/CD4-	CD38+HLA-DR+ activated T c/s	
	16.1	CD3	CD8+/CD4-	CD38+			CD3/CD8+/CD4-	CD38+ activated T c/s	
	17.1	CD3	CD8+/CD4-	HLA-DR+			CD3/CD8+/CD4-	HLA-DR+ activated T c/s	
	18	CD3	CD8+/CD4-	CD45RA+			CD3/CD8+/CD4-	CD45RA+ T c/s	
	19	CD3	CD8+/CD4-	CD45RA-			CD3/CD8+/CD4-	CD45RA- T c/s	
	20	CD3	CD8+/CD4-	CD28-	CCR7+/CD45RA+		CD3/CD8+/CD4-/CD28-	naïve T c/s	CD45RA+CCR7+ T c/s
	21	CD3	CD8+/CD4-	CD28-	CCR7+/CD45RA-		CD3/CD8+/CD4-/CD28-	central memory T c/s	CD45RA-CCR7+ T c/s
	22	CD3	CD8+/CD4-	CD28-	CCR7-/CD45RA-		CD3/CD8+/CD4-/CD28-	effector memory T c/s	CD45RA-CCR7- T c/s
	23	CD3	CD8+/CD4-	CD28-	CCR7-/CD45RA+		CD3/CD8+/CD4-/CD28-	terminal effector T c/s	CD45RA+CCR7- T c/s
	67	CD3	CD4+/CD8-	CD28+			CD3/CD4+/CD8-	CD28+ T helper	
	68	CD3	CD4+/CD8-	CD28-			CD3/CD4+/CD8-	CD28- T helper	
T helper	1.2	CD3					viable mononuclear cells	T cells	CD3+ viable mononuclear cells
	2.2	CD3	CD4+/CD8+				CD3	DP T cells	CD4+CD8+ T cells
	3.2	CD3	CD4-/CD8-				CD3	DN T cells	CD4-CD8- T cells
	4.2	CD3	CD4+/CD8-				CD3	T helper	CD4+CD8- T cells
	5.2	CD3	CD4+/CD8-	HLA-DR+/CD38+			CD3/CD4+/CD8-	CD38+HLA-DR+ activated T helper	
	6.2	CD3	CD4+/CD8-	CD38+			CD3/CD4+/CD8-	CD38+ activated T helper	
	7.2	CD3	CD4+/CD8-	HLA-DR+			CD3/CD4+/CD8-	HLA-DR+ activated T helper	
	14.2	CD3	CD8+/CD4-				CD3/CD4-/CD8+	T c/s	CD4-CD8+ T cells
	15.2	CD3	CD8+/CD4-	HLA-DR+/CD38+			CD3/CD4-/CD8+	CD38+HLA-DR+ activated T c/s	
	16.2	CD3	CD8+/CD4-	CD38+			CD3/CD4-/CD8+	CD38+activated T c/s	
	17.2	CD3	CD8+/CD4-	HLA-DR+			CD3/CD4-/CD8+	HLA-DR+ activated T c/s	
	24	CD3	CD4+/CD8-	CXCR5+			CD3/CD4+/CD8-	Tfh	CXCR5+ T helper
	25	CD3	CD4+/CD8-	CXCR3+/CCR6-			CD3/CD4+/CD8-	Th1	CXCR3+CCR6- T helper
	26	CD3	CD4+/CD8-	CXCR3-/CCR6-			CD3/CD4+/CD8-	Th2	CXCR3-CCR6- T helper
	27	CD3	CD4+/CD8-	CXCR3-/CCR6+			CD3/CD4+/CD8-	Th17	CXCR3-CCR6+ T helper
	28	CD3	CD8+/CD4-	CXCR5+			CD3/CD4-/CD8+	CXCR5+ Tc/s	CXCR5+ Tc/s
	29	CD3	CD8+/CD4-	CXCR3+/CCR6-			CD3/CD4-/CD8+	Tc1	CXCR3+CCR6- T c/s
	30	CD3	CD8+/CD4-	CXCR3-/CCR6-			CD3/CD4-/CD8+	Tc2	CXCR3-CCR6- T c/s
	31	CD3	CD8+/CD4-	CXCR3-/CCR6+			CD3/CD4-/CD8+	Tc17	CXCR3-CCR6+ T c/s
T-reg	1.3	CD3					viable mononuclear cells	T cells	CD3+ viable mononuclear cells
	32	CD3	CD4+				CD3	T helper	CD4+ T cells
	33	CD3	CD4+	HLA-DR+/CD39+			CD3/CD4+	CD39+ HLA-DR+ activated T helper	
	34	CD3	CD4+	HLA-DR+			CD3/CD4+	HLA-DR+ activated T helper	
	35	CD3	CD4+	CD39+			CD3/CD4+	CD39+ activated T helper	
	36	CD3	CD4+	CD127lo/CD25+			CD3/CD4+	Treg	CD127lowCD25+ T helper
	37	CD3	CD4+	CD127lo/CD25+	CCR4-/CD45RO+		CD3/CD4+/CD127lo/CD25+	CD127lo CD25+ CCR4- memory T cell	
	38	CD3	CD4+	CD127lo/CD25+	CCR4+/CD45RO-		CD3/CD4/CD127lo/CD25+	naïve Treg	CD45RO-CCR4+ Treg
	39	CD3	CD4+	CD127lo/CD25+	CCR4+/CD45RO+	HLA-DR+	CD3/CD4/CD127lo/CD25+/CCR4+/CD45RO+	HLA-DR+ memory Treg	
	40	CD3	CD4+	CD127lo/CD25+	CCR4+/CD45RO-	HLA-DR+	CD3/CD4/CD127lo/CD25+/CCR4+/CD45RO-	HLA-DR+ naïve Treg	

Table S4 continued.

Tube	Pop Code	Gate 1	Gate 2	Gate 3	Gate 4	Gate 5	Parent population for expressing frequency of defined population	Subset name	Subset name 2
B-cell	41	CD3-/CD19+					viable mononuclear cells	CD19+ B cells	CD19+CD3- viable mononuclear cells
	42	CD3-/CD20+					viable mononuclear cells	CD20+ mature B cells	CD20+CD3- viable mononuclear cells
	43	CD19+/CD20+					viable mononuclear cells	CD19+CD20+ B cells	CD19+CD20+ viable mononuclear cells
	44.1	CD19+/CD20+	CD10+/CD27lo				CD19+/CD20+	transitional B cells	CD10+CD27lo CD19+CD20+ B cells
	44.2	CD19+/CD20+	CD24hi/CD38hi				CD19+/CD20+	transitional B cells	CD24hiCD38hi CD19+CD20+ B cells
	44.3	CD19+/CD20+	CD24hi/CD10+				CD19+/CD20+	transitional B cells	CD10+CD24hi CD19+CD20+ B cells
	45	CD19+/CD20+	CD27-/IgD+				CD19+/CD20+	CD27-IgD+ naïve B cells	CD27-IgD+ CD19+CD20+ B cells
	46	CD19+/CD20+	CD27-/IgD-				CD19+/CD20+	CD27-IgD- naïve B cells	CD27-IgD- CD19+CD20+ B cells
	47	CD19+/CD20+	CD27+/IgD+				CD19+/CD20+	CD27+IgD+ memory B cells	CD27+IgD+ CD19+CD20+ B cells
	48	CD19+/CD20+	CD27+/IgD-				CD19+/CD20+	CD27+IgD- memory B cells	CD27+IgD- CD19+CD20+ B cells
	49	CD19+/CD20+	CD27-/IgD+	CD38hi			CD19+/CD20+/CD27-/IgD+	CD38high activated CD27-IgD+ naïve B cells	
	50	CD19+/CD20+	CD27-/IgD-	CD38hi			CD19+/CD20+/CD27-/IgD-	CD38high activated CD27-IgD- naïve B cells	
	51	CD19+/CD20+	CD27+/IgD+	CD38hi			CD19+/CD20+/CD27+/IgD+	CD38high activated CD27+IgD+ memory B cells	
	52	CD19+/CD20+	CD27+/IgD-	CD38hi			CD19+/CD20+/CD27+/IgD-	CD38high activated CD27+IgD- memory B cells	
	53	CD19+/CD20-					viable mononuclear cells	CD20- B cells	CD19+CD20- viable mononuclear cells
	54.1	CD19+/CD20-	CD27hi/CD38hi				CD19+CD3-	plasmablasts as fraction of all B cells	CD27hiCD38hi CD20- B cells as fraction of all B cells
	54.2	CD19+/CD20-	CD27hi/CD38hi				CD19+/CD20-	plasmablasts as fraction of CD20- B cells	CD27hiCD38hi CD20- B cells as fraction of CD20- B cells
	69	CD19+/CD20+	CD38high				CD19+/CD20+	CD38high activated B cells	
DC Mono NK	55	Lin-/CD14+					viable mononuclear cells	Monocytes	CD14+ viable mononuclear cells
	56	Lin-/CD14+	CD16-				Lin-/CD14+	classical monocytes	CD14+CD16- monocytes
	57	Lin-/CD14+	CD14dim/CD16+				Lin-/CD14+	non-classical monocytes	CD14dimCD16+ monocytes
	58	Lin-/CD14+	HLA-DR+ (MFI)				Lin-/CD14+	monocytes, HLA-DR antigen density	monocyte HLA-DR MFI
	59	Lin-/CD14-	CD16+/CD56-				Total NK (CD56+ and CD56-CD16+)	CD16+CD56- NK cells	
	60	Lin-/CD14-	CD16+/CD56+				Total NK (CD56+ and CD56-CD16+)	CD16+CD56+ NK cells	
	61	Lin-/CD14-	CD16-/CD56+				Total NK (CD56+ and CD56-CD16+)	CD16-CD56+ NK cells	
	62	Lin-/CD14-	CD16+/CD56-	HLA-DR+			Lin-/CD14-/CD16+/CD56-	HLA-DR+ activated CD16+CD56- NK cells	
	63	Lin-/CD14-	CD16+/CD56+	HLA-DR+			Lin-/CD14-/CD16+/CD56+	HLA-DR+ activated CD16+CD56+ NK cells	
	64	Lin-/CD14-	CD16-/CD56+	HLA-DR+			Lin-/CD14-/CD16-/CD56+	HLA-DR+ activated CD16-CD56+ NK cells	
	65	Lin-/CD14-	CD16-/CD56-	HLA-DR+	CD11c+/CD123-		Lin-/CD14-/CD16-/CD56-/HLA-DR+	myeloid dendritic cells	CD11c+CD123- HLA-DR+ CD16-CD56- Lin-CD14- cells
	66	Lin-/CD14-	CD16-/CD56-	HLA-DR+	CD123+/CD11c-		Lin-/CD14-/CD16-/CD56-/HLA-DR+	plasmacytoid dendritic cells	CD123+CD11c- HLA-DR+ CD16-CD56- Lin-CD14- cells

Table S5. Panels of serum proteins analyzed by Luminex. Related to STAR Methods.

**Kit name**	**cytokine grp I**	**cytokine grp II**	**Diabetes**	**Th17**	**Acute phase**	**Acute phase**
**number of analytes**	**27**	**21**	**10**	**9**	**5**	**4**
Dilution factor	**4**	**4**	**4**	4	**1000**	**10000**
**analyte (bead region)**	PDGF-bb (47)	CTACK (72)	C-peptide (72)	IL-17A (76)	PCT (56)	A2M (92)
	IL-1b (39)	GROa (61)	Ghrelin (26)	IL-17F (44)	Ferritin (38)	Haptoglobin (79)
	IL-1ra (25)	IL-1a (63)	GIP (14)	IL-21 (47)	tPA (21)	CRP (81)
	IL-2 (38)	IL-2Ra (13)	GLP-1 (27)	IL-22 (18)	Fibrinogen (52)	SAP (98)
	IL-4 (52)	IL-3 (64)	Glucagon (15)	IL-23 (43)	SAA (96)	
	IL-5 (33)	IL-12p40 (28)	Insulin (12)	IL-25 (55)		
	IL-6 (19)	IL-16 (27)	Leptin (78)	IL-31 (62)		
	IL-7 (74)	IL-18 (42)	PAI-1 (61)	IL-33 (46)		
	IL-8 (54)	LIF (29)	Resistin (65)	sCD40L (29)		
	IL-9 (77)	MCP-3 (26)	Visfatin (22)			
	IL-10 (56)	M-CSF (67)				
	IL-12(p70) (75)	MIF (35)				
	IL-13 (51)	MIG (14)				
	IL-15 (73)	b-NGF (46)				
	IL-17A (76)	SCF (65)				
	Eotaxin (43)	SCGF-b (78)				
	FGF basic (44)	SDF-1a (22)				
	G-CSF (57)	TNF-b (30)				
	GM-CSF (34)	TRAIL (66)				
	IFN-g (21)	HGF (62)				
	IP-10 (48)	IFN-a2 (20)				
	MCP-1 (53)					
	MIP-1a (55)					
	MIP-1b (18)					
	RANTES (37)					
	TNF-a (36)					
	VEGF (45)					

Data S1. Analysis code workflow. Related to STAR Methods.

1 Table of Contents

2 Environment Setup 3
2.1 Singularity Container 3
3 Titers and Pattern Profiles of Clinical CBC and Luminex Data 3
3.1 Figure 1 3
3.2 Supplemental Figure 9 3
4 Gene Expression (PBMC) Data Processing 4
4.1 Expression Set Creation from APT Output 4
4.2 Probesets to Genes Mapping 4
4.2.1 Convert the Table to Probeset-Gene Mapping 4
4.2.2 Select the Best Probeset for a Gene 4
4.3 Data Post Processing 4
4.3.1 Correct the Switched Samples 4
4.3.2 Apply Different Filtering to Samples and Genes 4
4.3.3 Calculate Fold Change from Day 0 4
5 Pattern Discovery in Post-Vaccination Profiles of PBMC Gene Expression Data 5
5.1 Profiles Clustering with DIANA 5
5.2 Stable Cluster/Pattern Detection by Tree Cutting 5
5.3 Pattern Filtering 5
5.4 Patterns Stats Summary 5
5.5 Figure 2A 5
5.6 Figure 2B 5
5.7 Expanding the List of Pattern Signature Genes 5
5.8 Compute Correlations and Clean up Gene List 5
5.8.1 Supplemental Figure 1A 5
5.9 Compute Subject Scores for Each Pattern 6
5.10 Table of Genes (with annotations) for Each Pattern 6
5.11 Figure 2C 6
5.12 Add Data for Subject s10 and Update the Score Matrix 6
6 Pattern Discovery in Post-Vaccination Profiles of Flow Cytometry Data 6
6.1 Generate Trajectory Matrix 6
6.2 Generate Trajectory Clusters 6
6.3 Figure 2D, 2E, and 2F 6
6.4 Supplemental Figure 2 7
7 Find Signature for Adjuvant Status Prediction using PBMC Data 7

7.1 FIGURE 3C 7
7.2 FIGURE 3D 7
7.3 SUPPLEMENTAL FIGURE 3B 7
7.4 FIGURE 3E 7
7.5 ELASTIC NET MODELS 7
7.5.1 GENERATE INPUT DATA 7
7.5.2 RUN ENETXPLORER 7
7.5.3 FIGURE 3B 7
7.5.4 FIGURE 3G, AND 3H 8
8 GENE EXPRESSION (WHOLE BLOOOD/PAXGENE) DATA PROCESSING 8
8.1 DATA POST PROCESSING 8
8.2 APPLY DIFFERENT FILTERING TO SAMPLES AND GENES 8
8.3 CALCULATE FOLD CHANGE FROM DAY 0 8
9 UNBLINDING ADJUVANT STATUS 8
9.1 SUPPLEMENTAL FIGURE 4A 8
10 BLINDLY PREDICTING ADJUVANT STATUS USING DATA FROM EMORY UNIVERSITY 8
10.1 PROCESS DATA TO GENERATE ESPRESSION SET 8
10.2 GET ANNOTATIONS 9
10.3 MAP PROBES TO GENES 9
10.4 CALCULATE 2 PEAK SCORES 9
10.5 SUPPLEMENTAL FIGURE 4B & 4C 9
11 DAY 100 CITE-SEQ ANALYSIS 9
11.1 MICROARRAY DIFFERENTIAL EXPRESSION OF PERSISTENCE PATTERNS; DAY 100 VS. BASELINE RANDOM INTERCEPT MODEL 9
11.2 CITE-SEQ DAY 100 NORMALIZATION AND PROTEIN BASED CLUSTERING 10
11.2.1 CITE-SEQ DIFFERENTIAL EXPRESSION ANALYSIS AND ENRICHMENT OF PERSISTENCE GENES WITHIN CELL TYPES 10
11.2.2 TEST LEADING EDGE GENES FROM SINGLE CELL DAY 100 H5N1 VACCINES IN H1N1 HIGH VS LOW RESPONDERS 10
11.3 ANALYSIS OF PERSISTENCE SIGNALS IN H1N1 VACCINE DAY 70 VS BASELINE 10
11.4 CITE-SEQ ANALYSIS OF PERSISTENCE SIGNALS IN H5N1 VACCINE DAY 100 VS DAY 21 10
12 ATAC-SEQ ANALYSIS 11
12.1 MOTIF ENRICHMENT 11
12.2 PLOTTING 11

2 Environment Setup

2.1 Singularity Container

The Singularity container (h5n1_workflow.sif) contains the specific R version and the packages used for data analysis from section 3 to section 12. Folder paths are relative to the project root folder.

To run a script using the container (put the container inside the project's root folder):

```
$ singularity exec h5n1_workflow.sif Rscript –vanilla <script path>
```

3 Titers and Pattern Profiles of Clinical CBC and Luminex Data

3.1 Figure 1

B.

```
Rscript --vanilla SCRIPTS/titers/mn_titer_profiles.r
```

C.

```
Rscript --vanilla SCRIPTS/profiles/Monocytes_figure.r
```

D.

```
Rscript --vanilla SCRIPTS/profiles/Neutrophils_figure.r
```

E.

```
Rscript --vanilla SCRIPTS/profiles/IP10_figure.r
```

3.2 Supplemental Figure 9

A.

```
Rscript --vanilla SCRIPTS/titers/titer_response_rate.r
```

B.

```
Rscript --vanilla SCRIPTS/titers/hai_titer_profiles.r
```

C.

```
Rscript --vanilla SCRIPTS/titers/mn_titer_peak.r
```

4 Gene Expression (PBMC) Data Processing

First, the CEL files were processed using Affymetrix power tools.

```
Rscript --vanilla  SCRIPTS/MA/processing_pbmc/apt.config.r
Rscript --vanilla SCRIPTS/MA/processing_pbmc/apt.call.r
```

Note: Power Tools are not included in the singularity container.

4.1 Expression Set Creation from APT Output

```
Rscript --vanilla SCRIPTS/MA/processing_pbmc/eset.config.r
Rscript --vanilla SCRIPTS/MA/processing_pbmc/eset.call.r
```

4.2 Probesets to Genes Mapping

4.2.1 Convert the Table to Probeset-Gene Mapping

```
Rscript --vanilla SCRIPTS/MA/annotation/affy_hugene-2_1-st_annotation.r
```

4.2.2 Select the Best Probeset for a Gene

```
Rscript --vanilla SCRIPTS/MA/annotation/generate_ps2gene_map.config.r
Rscript --vanilla SCRIPTS/MA/annotation/generate_ps2gene_map.call.r
```

4.3 Data Post Processing

4.3.1 Correct the Switched Samples

```
Rscript --vanilla SCRIPTS/MA/filtering_pbmc/switch.samples/switch.samples.call.r
```

4.3.2 Apply Different Filtering to Samples and Genes

```
Rscript --vanilla SCRIPTS/MA/filtering_pbmc/samples.clean_genes.all/filtering.r
Rscript --vanilla SCRIPTS/MA/filtering_pbmc/samples.clean_genes.iqr/filtering.r
Rscript --vanilla SCRIPTS/MA/filtering_pbmc/samples.all_genes.all/filtering.r
Rscript --vanilla SCRIPTS/MA/filtering_pbmc/samples.all_genes.iqr/filtering.r
```

4.3.3 Calculate Fold Change from Day 0

```
Rscript --vanilla SCRIPTS/MA/calculate_d0_fc/calculate_d0_fc_pbmc.r
```

5 Pattern Discovery in Post-Vaccination Profiles of PBMC Gene Expression Data

5.1 Profiles Clustering with DIANA

```
Rscript --vanilla SCRIPTS/MA/pattern_discovery/pattern_discovery.r
```

5.2 Stable Cluster/Pattern Detection by Tree Cutting

```
Rscript --vanilla SCRIPTS/MA/pattern_discovery/patterns_cutTree_stable.r
```

5.3 Pattern Filtering

```
Rscript --vanilla SCRIPTS/MA/pattern_discovery/pattern_filter.r
```

5.4 Patterns Stats Summary

```
Rscript --vanilla SCRIPTS/MA/pattern_discovery/patterns_stats.r
```

5.5 Figure 2A

```
Rscript --vanilla SCRIPTS/pattern_sim/pattern_simulation.r
```

5.6 Figure 2B

Patterns Profile Plot

```
Rscript --vanilla SCRIPTS/MA/pattern_discovery/plot_patterns.r
```

5.7 Expanding the List of Pattern Signature Genes

```
Rscript --vanilla SCRIPTS/MA/pattern_discovery/pattern_filter_expanded.r
```

5.8 Compute Correlations and Clean up Gene List

5.8.1 Supplemental Figure 1A

```
Rscript --vanilla SCRIPTS/MA/pattern_discovery/pattern_expanded_genes_cor.r
Rscript --vanilla SCRIPTS/MA/pattern_discovery/pattern_expanded_genes_clean.r
```

5.9 Compute Subject Scores for Each Pattern

```
Rscript --vanilla SCRIPTS/MA/pattern_discovery/patterns_to_subjects.r
```

5.10 Table of Genes (with annotations) for Each Pattern

```
Rscript --vanilla SCRIPTS/MA/pattern_discovery/pattern_genes_output.r
```

5.11 Figure 2C

BTM Enrichment In Patterns Genes

```
Rscript --vanilla SCRIPTS/MA/pattern_discovery/pattern_BTM_enrichment.r
```

5.12 Add Data for Subject s10 and Update the Score Matrix

```
Rscript --vanilla SCRIPTS/MA/pattern_discovery/s10_peaks_assessment.r
Rscript --vanilla
SCRIPTS/MA/pattern_discovery/pattern_scores_in_samples_GE_incl.s10.r
```

6 Pattern Discovery in Post-Vaccination Profiles of Flow Cytometry Data

FlowJo software was used to export flow data.

6.1 Generate Trajectory Matrix

```
Rscript --vanilla SCRIPTS/Flow_10c/Flow_10c_TrajMatrix.R
```

6.2 Generate Trajectory Clusters

```
Rscript --vanilla SCRIPTS/Flow_10c/Flow_10c_TrajCluster.R
```

6.3 Figure 2D, 2E, and 2F

D.

```
Rscript --vanilla SCRIPTS/Flow/pattern_figures/plot_flow_patters_only.r
```

E.

```
Rscript --vanilla SCRIPTS/Flow/pattern_figures/pattern_flow_ann_heatmap.r
```

F.

```
Rscript --vanilla SCRIPTS/MA/pattern_discovery/pattern_scores_GE_flow_heatmap.r
```

6.4 Supplemental Figure 2

```
Rscript --vanilla SCRIPTS/Flow_10c/Flow_10c_TrajCluster_QM.R
```

7 Find Signature for Adjuvant Status Prediction using PBMC Data

7.1 Figure 3C

```
Rscript --vanilla SCRIPTS/adjuvant_prediction/2peaks_pca_2clusters.r
```

7.2 Figure 3D

```
Rscript --vanilla SCRIPTS/adjuvant_prediction/ip10_2clusters_compare.r
```

7.3 Supplemental Figure 3B

```
Rscript --vanilla SCRIPTS/adjuvant_prediction/cytokines_2clusters_compare.r
```

7.4 Figure 3E

```
Rscript --vanilla SCRIPTS/adjuvant_prediction/ip10_2peak_scores.r
Rscript --vanilla SCRIPTS/adjuvant_prediction/2peaks_pca_final_heamap.r
```

7.5 Elastic Net Models

7.5.1 Generate Input Data

```
Rscript --vanilla SCRIPTS/eNetXplorer/eNet_input_r1.r
Rscript --vanilla SCRIPTS/eNetXplorer/eNet_input_r2.r
Rscript --vanilla SCRIPTS/eNetXplorer/eNet_input_r3.r
```

7.5.2 Run eNetXplorer

```
Rscript --vanilla SCRIPTS/eNetXplorer/eNetXplorer_R1_180530.R
Rscript --vanilla SCRIPTS/eNetXplorer/eNetXplorer_R2_180530.R
Rscript --vanilla SCRIPTS/eNetXplorer/eNetXplorer_R3_180530.R
```

7.5.3 Figure 3B

```
Rscript --vanilla SCRIPTS/eNet_figures/enet_plots_R1.r
```

7.5.4 Figure 3G, and 3H

G.

```
Rscript --vanilla SCRIPTS/eNet_figures/enet_plots_R3.r
```

H.

```
Rscript --vanilla SCRIPTS/eNet_figures/enet_plots_R2.r
```

8 Gene Expression (Whole Bloood/PAXgene) Data Processing

8.1 Data Post Processing

Note: We found that two samples were switched. This is to correct it.

```
Rscript --vanilla SCRIPTS/MA/filtering_pax/switch.samples/switch.samples.call.r
```

8.2 Apply Different Filtering to Samples and Genes

```
Rscript --vanilla SCRIPTS/MA/filtering_pax/filtering.r
```

8.3 Calculate Fold Change from Day 0

```
Rscript –vanilla SCRIPTS/MA/calculate_d0_fc/calculate_d0_fc_pax.r
```

9 Unblinding Adjuvant Status

9.1 Supplemental Figure 4A

```
Rscript --vanilla SCRIPTS/adjuvant_prediction/pattern_gene_time_score_sel.subject.r
Rscript --vanilla SCRIPTS/adjuvant_prediction/pattern_flow_time_score_sel.subject.r
Rscript --vanilla SCRIPTS/adjuvant_prediction/IP10_time_score_sel.subject.r
```

10 Blindly Predicting Adjuvant Status using Data from Emory University

10.1 Process Data to Generate Espression Set

```
Rscript --vanilla SCRIPTS/Emory/emory_data.r
```

10.2 Get Annotations

```
Rscript --vanilla SCRIPTS/Emory/get_ann.r
```

10.3 Map Probes to Genes

```
Rscript --vanilla SCRIPTS/Emory/probe2gene.r
```

10.4 Calculate 2 Peak Scores

```
Rscript --vanilla SCRIPTS/Emory/2peak_scores.r
```

10.5 Supplemental Figure 4B & 4C

```
Rscript --vanilla SCRIPTS/Emory/adjuvant_prediction.r
Rscript --vanilla SCRIPTS/MA/baseline/analyze_IFNg_genes_emory_171208.r
Rscript --vanilla SCRIPTS/Emory/emory_pattern_scores_wrong_subjects.r
Rscript --vanilla SCRIPTS/Emory/ emory_ip10_wrong_sbujects.r
```

11 Day 100 CITE-Seq Analysis

Scripts in this section were used to generate sub-panels of Figure 4 and Supplementary Figures 7 and 8.

To run analysis, install selected functions from scglmmr R package by installing the included (in the root folder of the repository) package file locally.

```
install.packages(file.path(path_to_package,"scglmmr"), repos = NULL, type =
"source")
```

Note: for the subsequent scripts change to "H5_d100_public-master" folder in the root folder of the repository. These scripts are not part of the singularity container and folder structure mentioned above.

11.1 Microarray Differential Expression of Persistence Patterns; day 100 vs. Baseline Random Intercept Model

Note: Microarray analysis section uses R 3.6.1.

Format Data

```
source("microarray_analysis_d100/1_save_eset_object_as_dataframe.r")
```

Variance Partition and Mixed GLM Workflow

```
source("microarray_analysis_d100/2_V4array_d100_lme4_PERSISTENCE_V4.R")
```

Run Separate Models for Adjuvant and Non-Adjuvant Group

```
source("microarray_analysis_d100/2_array_adjonly_d100_lme4_PERSISTENCE_V3.R")
```

```
source("microarray_analysis_d100/2_nonadj_array_d100_lme4_PERSISTENCE_V3.R")
```

Save Results Table Combined

```
source("microarray_analysis_d100/3_make_combined_table.r")
```

11.2 CITE-seq day 100 Normalization and Protein Based Clustering

Note: Single cell analysis section uses R 3.5.3.

Normalize Single Cells from Baseline and Day 100, Cluster Annotate and run UMAP

```
source("2_clustering/3_umap.r")
```

11.2.1 CITE-seq Differential Expression Analysis and Enrichment of Persistence Genes Within Cell Types

Pseudobulk Workflow

```
source("3_pseudobulk_de_workflow/2_h5_d0_vd_d100_pseudobulk_de.r")
```

```
source("3_pseudobulk_de_workflow/3_figure_generation_h5d100cite_scglmmrv2.r")
```

11.2.2 Test Leading Edge Genes from Single Cell day 100 H5N1 Vaccines in H1N1 High vs Low Responders

```
source("3_pseudobulk_de_workflow/4_h1_highresponder_baseline_persistencesig_test.r"
)
```

```
source("3_pseudobulk_de_workflow/5_h1_highresponder_baselinepersistence_figure_gene
ration.r")
```

11.3 Analysis of Persistence Signals in H1N1 Vaccine day 70 vs Baseline

```
source("d70_H1/day70_h1_persistencegenes.r")
```

11.4 CITE-seq Analysis of Persistence Signals in H5N1 Vaccine day 100 vs day 21

```
Rscript --vanilla CITE-seq_day100_v_day21/code_foo_h5.R
```

12 ATAC-Seq Analysis

Scripts in this section were used to generate sub-panels of Figure 4 and Supplementary Figure 7.

Code and associated data folder: ATAC-seq

The code in 01_archr is provided for reference but won't be run as the raw data is being used in another forthcoming publication
The workflow starts after the 02_chromvar_pseudobulk_de/0_*_pool_and_limma.R scripts. Two files need to be present in 02_chromvar_pseudobulk_DE/outs/ :

```
02_chromvar_pseudobulk_DE/outs/CD14_Mono/cd14_mono_pbulk_list.rds
02_chromvar_pseudobulk_DE/outs/CD8_Naive/cd8_naive_pbulk_list.rds
```

Then do:

```
Rscript 02_chromvar_pseudobulk_DE/cd8naive_dream_adjuvant_contrast.R
Rscript 02_chromvar_pseudobulk_DE/cd14_mono_dream_adjuvant_contrast.R
```

Using singularity container pulled with: singularity pull docker://hukai916/r_sc:0.5

```
Singularity shell r_sc:0.5
Rscript 03_motif_enrichment_prep/1_cd14mono_subset_grange_hits.R
Rscript 03_motif_enrichment_prep/1_cd8naive_subset_grange_hits.R
Rscript 03_motif_enrichment_prep/2_cd14mono_leunion_grange_fasta.R
Rscript 03_motif_enrichment_prep/2_cd8naive_leunion_grange_fasta.R
```

12.1 Motif Enrichment

Make sure that file below is present
04_motif_enrichment_centrimo/motif_databases/HUMAN/HOCOMOCOv11_core_HUMAN_mono_meme_format.meme

```
bash http://04_motif_enrichment_centrimo/1_run_centrimo_cd8naive.sh
bash http://04_motif_enrichment_centrimo/1_run_centrimo.sh
```

12.2 Plotting

```
Singularity shell r_sc:0.5
Rscript 05_paper_figures/cd8_naive_plot_adjuvant_contrasts.R
Rscript 05_paper_figures/cd14_mono_plot_adjuvant_contrasts.R
```
